# Characterization and dark oxidation of the emissions of a pellet stove†

**DOI:** 10.1039/d3ea00070b

**Published:** 2023-07-28

**Authors:** Kalliopi Florou, John K. Kodros, Marco Paglione, Spiro Jorga, Stefania Squizzato, Mauro Masiol, Petro Uruci, Athanasios Nenes, Spyros N. Pandis

**Affiliations:** a Institute of Chemical Engineering Sciences, ICE-HT Patras 26504 Greece; b Institute of Atmospheric Sciences and Climate, Italian National Research Council Bologna 40129 Italy; c Department of Chemical Engineering, Carnegie Mellon University Pittsburgh 15213 USA; d Department of Environmental Sciences, Informatics and Statistics, Università Ca' Foscari Venezia Venice Italy; e Department of Chemical Engineering, University of Patras Patras 26504 Greece spyros@chemeng.upatras.gr; f School of Architecture, Civil and Environmental Engineering, Swiss Federal Institute of Technology Lausanne Lausanne 1015 Switzerland

## Abstract

Pellet combustion in residential heating stoves has increased globally during the last decade. Despite their high combustion efficiency, the widespread use of pellet stoves is expected to adversely impact air quality. The atmospheric aging of pellet emissions has received even less attention, focusing mainly on daytime conditions, while the degree to which pellet emissions undergo night-time aging as well as the role of relative humidity remain poorly understood. In this study, environmental simulation chamber experiments were performed to characterize the fresh and aged organic aerosol (OA) emitted by a pellet stove. The fresh pellet stove PM_1_ (particulate matter with an aerodynamic diameter less than 1 μm) emissions consisted mainly of OA (93 ± 4%, mean ± standard deviation) and black carbon (5 ± 3%). The primary OA (POA) oxygen-to-carbon ratio (O : C) was 0.58 ± 0.04, higher than that of fresh logwood emissions. The fresh OA at a concentration of 70 μg m^−3^ (after dilution and equilibration in the chamber) consisted of semi-volatile (68%), low and extremely low volatility (16%) and intermediate-volatility (16%) compounds. The oxidation of pellet emissions under dark conditions was investigated by injecting nitrogen dioxide (NO_2_) and ozone (O_3_) into the chamber, at different (10–80%) relative humidity (RH) levels. In all dark aging experiments secondary organic aerosol (SOA) formation was observed, increasing the OA levels after a few hours of exposure to NO_3_ radicals. The change in the aerosol composition and the extent of oxidation depended on RH. For low RH, the SOA mass formed was up to 30% of the initial OA, accompanied by a moderate change in both O : C levels (7–8% increase) and the OA spectrum. Aging under higher RH conditions (60–80%) led to a more oxygenated aerosol (increase in O : C of 11–18%), but only a minor (1–10%) increase in OA mass. The increase in O : C at high RH indicates the importance of heterogeneous aqueous reactions in this system, that oxidize the original OA with a relatively small net change in the OA mass. These results show that the OA in pellet emissions can chemically evolve under low photochemical activity (*e.g.* the wintertime period) with important enhancement in SOA mass under certain conditions.

## Abstract 2

Pellet combustion in residential heating stoves has increased globally during the last decade. In this study, environmental simulation chamber experiments were performed to characterize the fresh and aged organic aerosol emitted by a pellet stove.

## Graphical abstract

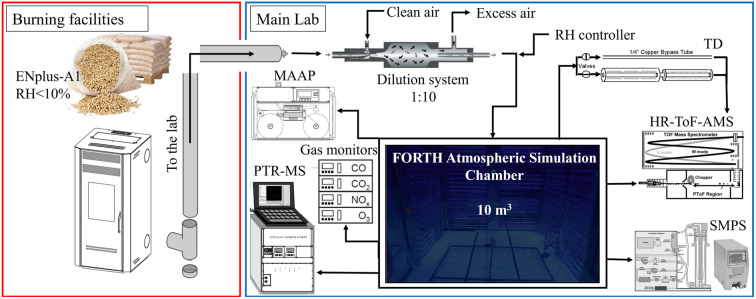

**Environmental significance**

Pellet combustion in residential heating stoves has increased globally during the last decade. However, this potentially important air pollution source has received relatively little attention compared to other sources of biomass burning. In this work we show that the corresponding emissions evolve chemically as they react during the night and day and that the corresponding production of secondary organic aerosol is quite different from that of logwood combustion. These results indicate that the emissions from pellet combustion need to be separated from the rest of the residential heating emissions and should be treated differently in chemical transport models. The characterization results (emission rates, composition, volatility distribution, *etc.*) can be used for the simulation of these emissions in the same models.

## 1. Introduction

Growing energy demand and recognition of the climatic effects of fossil fuel combustion have shifted interest towards alternative energy sources such as biomass combustion.^1^ During economic crises the consumption of wood for heating purposes, mostly as logs but also as pellets, has increased in households even in the developed world. Modern pellet boilers and stoves are an alternative to conventional log wood stoves because of their easy and automated operation, as well as their combustion efficiency which can exceed 90%.^2,3^ Several previous studies have quantified and compared emissions from the two types of appliances for different fuel types and the role of burning conditions.^4–9^

During the wintertime wood burning is one of the most important sources of organic aerosol (OA). Emissions consist of both primary organic aerosol (POA) and organic vapors that can be oxidized producing secondary organic aerosol (SOA).^10–13^ Pellet stoves are typically considered low-emitting burners,^14–16^ since they emit less particulate matter (PM)^17,18^ and carbon monoxide (CO) than modern wood stoves.^4,19^ A growing influence of their emissions on air pollution is expected. In 2018, global wood pellet consumption increased by 130% compared to its 2013 levels, reaching 53 million tons.^2^ Half of this consumption took place in Europe (27 million tons; a 60% increase in 5 years), where pellet consumption, for heating purposes only, increased by 220% during the 2013–2018 period, accounting for 15.8 million tons in 2018.^2^ In 2019, European pellet consumption increased by 7%, an increase of 1.8 million tons in just one year.^20^

The fresh emissions of pellet stoves consist mainly of OA and black carbon (BC),^7^ carbon dioxide and monoxide, nitrogen oxides, and a wide range of volatile organic compounds (VOCs).^19,21^ Bertrand *et al.*^5^ reported that the dominant (up to 90%) PM component of fresh pellet emissions was BC (concentrations higher than 100 μg m^−3^). The oxygen-to-carbon ratio (O : C) of OA was in the range of 0.2–0.6,^5,7^ with fresh emitted particles having a mass distribution with a mode at around 0.1 μm.^4,22^ The pellet OA density was found to range between 1020 kg m^−3^ and 1350 kg m^−3^.^23,24^ Differences in the emissions of different pellet types,^8,25,26^ the importance of different pellet burner operations^7,27^ and the significance of having ideal and non-ideal burning conditions^28^ have been underlined in previous studies.

The atmospheric aging of pellet emissions has received relatively little attention. Few previous studies have investigated SOA formation using chambers or flow reactors focusing on the oxidation of pellet emissions in the presence of sunlight (*i.e.*, *via* the OH radical). Bertrand *et al.*^5^ found that the OA mass increased by a factor of 1.5 to 1.9, after simulating atmospheric aging using ultraviolet light (UV), while Corbin *et al.*^6^ reported only minor amounts of SOA production relative to their primary OA emission from modern pellet stoves. Heringa *et al.*^7^ examined the aging of the emissions during different burning phases of a pellet burner (stable burning and starting) concluding that the O : C of OA increased in all cases, but SOA formation was quite variable ranging from low to a factor of 3.3 of the fresh OA, depending on the burning phase. Reyes *et al.*^29^ compared firewood and pellet emissions and concluded that firewood combustion emissions are more rapidly oxidized.

Recently, there has been growing interest in exploring the extent of atmospheric processing of biomass burning emissions during the night-time.^30,31^ Hartikainen *et al.*^32^ reported substantial SOA production in laboratory experiments aging logwood combustion emissions under dark conditions. Kodros *et al.*^33^ showed that fresh emissions from wood burning exposed to NO_3_ radicals rapidly formed oxygenated OA (OOA) and organic nitrate compounds. The extent of oxidation was sensitive to relative humidity (RH) and consistently showed greater enhancement in the O : C ratio under high RH conditions, suggesting a sensitivity of the aging process to water vapor and/or aerosol liquid water content.^34–36^ Jorga *et al.*,^37^ using a dual chamber system and ambient air during the wintertime, observed rapid SOA formation corresponding to 20–70% of the pre-existing OA, with secondary organic nitrate accounting for up to 85% of total aerosol nitrate. The SOA produced in both studies was chemically similar to ambient observations of oxidized OA factors.^38–40^

Despite these previous studies, aging of pellet emissions in the absence of sunlight has received little or no attention. In this study, we performed environmental smog chamber experiments to characterize fresh pellet stove emissions using high commercially available fuel class quality fuel and a modern pellet unit. Pellet combustion was representative of residential heating in an urban environment. The oxidation of fresh pellet emissions under dark conditions was investigated by injecting different concentrations of both nitrogen dioxide (NO_2_) and ozone (O_3_), at low and high relative humidity levels. We compared these dark-aging experiments to 3 reference experiments: two experiments without any external initiation of oxidation and one experiment under exposure to UV lights. The results were additionally compared to those of logwood burning in an effort to distinguish the two biomass burning sources.

## 2. Experimental procedure

Environmental simulation chamber experiments were performed in the Foundation of Research and Technology-Hellas atmospheric simulation chamber (FORTH-ASC) to characterize fresh and aged pellet stove emissions. The FORTH-ASC is a 10 m^3^ Teflon reactor inside a 30 m^3^ temperature-controlled room with polished aluminum walls to increase light intensity. The vertical walls are covered with ultraviolet (UV) fluorescent lamps (Osram, L 36W/73), resulting in a *J*_NO_2__ of 0.59 min^−1^ when all lights are turned on.

Pellets of the highest commercially available fuel class quality (defined by ISO 17225-2: ENplus A1; RH < 10%) were burned in a pellet stove (Atena 80, with a heat output of 3–6.7 kW) operated in its automatic mode. Both stem conifer wood and chemically untreated by-products were used as raw materials for pellet production. The fuel consumption in our experiments was 1.1 kg h^−1^, which is approximately in the middle of the range for this stove. The wood pellets had a diameter of 6–8 mm and a length of 5–20 mm. Both the fuel and pellet stove are commercially available in the European market and represent characteristic fuel and heating devices used in Greece.

**Fig. 1:**
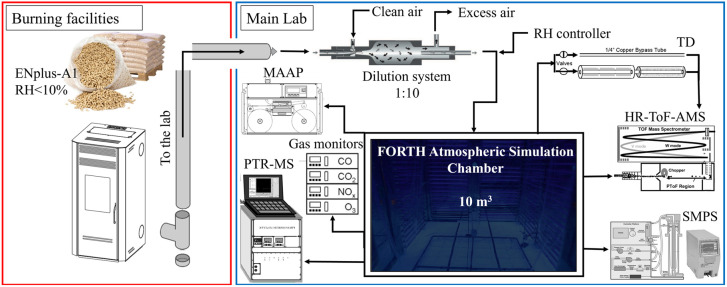
Schematic of the FORTH-ASC combustion chamber, pellet burning facilities and instrumentation.

The pellet combustion facility is located in the basement of the building, directly underneath the atmospheric simulation chamber (Fig. 1). In all experiments, we injected the pellet emissions into the chamber approximately 10–20 minutes after ignition under flaming conditions.

The pellet combustion emissions were directed into the environmental chamber, passing through a dilution system. The tubing used in the injection system was stainless steel (3/8 prior to the diluter and 1/2 after the diluter) and all lines were insulated. The corresponding selection was made to minimize losses of particles, but some adsorption of the gas-phase organics to the tubing can be expected. The flow was in the laminar regime. Emissions were diluted by approximately a factor of 10 with clean air. The chamber was pre-filled and the dilution in the chamber was close to 1 : 30. The target was an OA mass concentration in the range of 40–200 μg m^−3^, which corresponds to observed atmospheric OA levels during periods of intense residential biomass burning.

The fresh emissions were allowed to equilibrate in the dark chamber for approximately 2 hours to allow sufficient time for mixing and characterization. After this period, NO_2_ and O_3_ were injected in the dark. We define the injection of O_3_ as time zero for all dark-aging experiments. In the three reference experiments time zero is the time when the emissions were exposed to UV (Exp. 7) or when the pellet emissions were injected into the chamber without any extra addition of NO_2_ and O_3_ (Exp. 8 and 9; Table 1). Isotopically labelled butanol d-9 was added to the chamber to estimate OH levels.^41^ Prior to each experiment the chamber was cleaned overnight by flushing it with clean air.

**Table 1:** Experimental conditions for the dark, UV and blank experiments. All concentrations are those measured in the chamber after dilution

Exp.	Pellet burning emissions									Experimental conditions				
	OA [μg m^−3^]	BC [μg m^−3^]	Nitrate [μg m^−3^]	Total PM_1_ [μg m^−3^]	*f*_44_/*f*_60_	O : C	H : C	VOCs/NO_*x*_	MCE	Lights	RH [%]	*T* [°C]	O_3_ [ppb]	NO_2_ [ppb]
1	42	0.6	0.4	43.2	0.66	0.53	1.74	2.80	0.97	Dark	8	21	55	50
2	204	4.7	2	211.4	0.51	0.56	1.73	1.72	0.96	Dark	9	22	100	100
3	93	6.9	0.9	101.5	0.66	0.55	1.75	0.74	0.97	Dark	9	22	100	90
4	62	5.5	1.5	69.4	0.72	0.58	1.77	0.45	0.92	Dark	80	20	90	90
5	78	3	2.2	83.4	0.58	0.57	1.77	3.15	0.92	Dark	80	20	45	40
6	58	4.2	5	70.2	0.76	0.65	1.76	0.98	0.92	Dark	60	18	100	110
7	67	1.5	0.4	69.2	1.11	0.54	1.73	54.12	0.96	UV	10	22	(10)^a^	(0.3)^a^
8	84	2.6	1.8	90.6	1.57	0.57	1.69	6.50	0.93	Dark	15	20	(27)^a^	(4.5)^a^
9	74	4.2	0.9	81.9	0.62	0.64	1.80	5.77	0.96	Dark	9	22	(17)^a^	(4.7)^a^

a In experiments 7–9, no additional NO_2_ or O_3_ was injected.

We tried to quantify the extent of losses of semivolatile organic vapors in the chamber by allowing the emissions to remain undisturbed for up to 12 h. No changes in the particle composition in the chamber were observed using the AMS. Also the small changes in the OA mass concentration were consistent with the measured particle wall losses in the FORTH chamber. These observations indicate that the losses of semi-volatile and lower-volatility organics to the walls during our experiments were relatively low.

### 2.1 Instrumentation

A high-resolution time-of-flight aerosol mass spectrometer (HR-ToF-AMS, Aerodyne Research Inc., Billerica, USA)^42,43^ was used to provide continuous quantitative size and composition information for the non-refractory PM_1_ (particles with diameter less than 1 μm) aerosol in real time. In this study no drier was used prior to aerosol sampling. The vaporizer surface temperature was set at 600 °C and measurements were collected with a 3 min temporal resolution. Measurements from the HR-ToF-AMS were analyzed using the standard AMS software toolkits, SeQUential Igor data RetRiEvaL (SQUIRREL) v1.57I and Peak Integration by Key Analysis (PIKA) v1.16I within Igor Pro 6.37 (Wave Metrics). Elemental ratios, such as the oxygen-to-carbon (O : C) ratio were determined following the improved method by Canagaratna *et al.*^44^

Parallel to the AMS, a scanning mobility particle sizer (SMPS; classifier model 3080, DMA model 3081, CPC model 3787, TSI Incorporated) was used, with a sheath flow rate set at 3 L min^−1^ and an aerosol sample flow rate of 0.6 L min^−1^, measuring the particulate number size distribution for mobility diameters ranging between 14 and 710 nm every 3 min. A multiple-angle absorption photometer (MAAP, Thermo Scientific Inc.)^45^ was deployed to determine the BC levels. Due to its high flow (16.7 L min^−1^), the MAAP was sampling from the chamber for 10 min every half hour, with a time resolution of 1 min. A mass absorption cross section of BC of 6.6 m^2^ g^−1^ at a wavelength of 637 nm was assumed for the estimation of the BC concentration.

The OA volatility was measured using a thermodenuder (TD).^46,47^ The measurements were analyzed using the Karnezi *et al.*^48^ algorithm to estimate the volatility distribution of OA.^49^ The TD consists of a heated tube where more volatile compounds evaporate followed by a cooling section with activated carbon to avoid vapor condensation. The TD was combined with the SMPS and the AMS to measure the aerosol mass fraction remaining (MFR) in a selected range of temperatures. The mass transfer in TDs depends on the initial OA concentration, the residence time in the heating tube, the vaporization enthalpy of the OA, and any mass transfer resistances.

The concentration of VOCs was measured using a proton-transfer-reaction mass spectrometer (PTR-QMS 500, Ionicon Analytik). The reaction reagent for the PTR-QMS was H_3_O^+^. The drift tube was operated at 600 V at a constant pressure of 2.2–2.3 mbar and the sampling flow rate was 0.5 L min^−1^. The calibration followed the procedure described by Kaltsonoudis *et al.*^50^ using VOC standards obtained from Ionicon.

A suite of Teledyne gas monitors was used to monitor the concentration of nitrogen oxides and ammonia (T201), ozone (O_3_; 400E), carbon monoxide (CO; 300E), and carbon dioxide (CO_2_; T360). Gas monitors were routinely calibrated.

Sampling on 47 mm PTFE filters (Pall Corporation, 1 cm diameter of the collection surface) was performed at a flow rate of 8 L min^−1^ for 20 min for primary and aged PM_1_ pellet emissions in order to perform offline Fourier transform infrared spectroscopy (FTIR). Details about the FTIR analysis can be found in Yazdani *et al.*^51^

### 2.2 Data analysis and methods

For the determination of the AMS collection efficiency (CE) and the OA density, the algorithm of Kostenidou *et al.*^52^ was used, which compares the SMPS volume distributions and the AMS mass distributions. The BC measured using the MAAP was also taken into account in the algorithm, under the assumption that BC had the same size distribution as the OA, since the MAAP cannot provide this information.

The size-dependent particle wall loss rate constants were measured after each experiment using ammonium sulfate seeds.^53,54^ Particles were produced using an atomizer and after passing through a dryer they were introduced to the chamber directly. The corresponding wall loss rate constants were nearly constant across particle diameters in the range of 60–700 nm. We corrected AMS mass concentrations using the average wall loss rate constant (0.06 ± 0.02 h^−1^) at the mean vacuum aerodynamic diameter of the OA mass distribution (170 nm).

To explore the range of solutions of the TD model, we discretized the domain of the parameters and simulated all combinations of volatilities, the vaporization enthalpy (Δ*H*_vap_), and the accommodation coefficient (*α*_m_). We used 7 bins for the volatility ranging from 10^−3^ to 10^3^ μg m^−3^ and varied the mass fraction from 0 to 1 with a step of 0.1, leading to around 8000 different combinations. The values used for Δ*H*_vap_ ranged from 20 to 200 kJ mol^−1^ with a step of 20 kJ mol^−1^, and for *α*_m_, the discrete values are 0.001, 0.01, 0.1, and 1. Around 320 000 simulations were performed for all combinations of all properties, from which we identified those that led to errors less than 1%. Then we calculated the best estimate following the approach described by Karnezi *et al.*^48^ The one-dimensional volatility basis set (1 D-VBS) framework has been designed to help describe the volatility distribution of OA. The 1D-VBS utilizes logarithmically spaced bins based on an effective saturation concentration at 298 K.^55^

The theta angle (*θ*) was used to quantify the variation in the SOA mass spectra measured using the AMS.^56^ The theta angle is the inner product of mass spectra treated like *n*-dimensional vectors (where *n* is the number of mass-to-charge ratio, *m*/*z*, values). A theta angle of 10° or less indicates high similarity, while higher values (more than 25°) suggest significant differences between the two spectra.

Particulate organic nitrate (ON) was estimated based on the NO_2_^+^/NO^+^ ratio.^57–59^ In this work, the measured NO_2_^+^/NO^+^ ratio for pure ammonium nitrate (NH_4_NO_3_) was equal to 0.82, while the minimum observed NO_2_^+^/NO^+^ratio during all experiments was 0.04.

OH radical concentrations were estimated from the change in the concentration of the PTR-MS *m*/*z* 66 which corresponds to the isotopically labeled butanol-d9 (98%, Cambridge Isotope Laboratories). The corresponding reaction rate constant used was 3.4 × 10^−12^ cm^3^ per molecule per s.^41^ One photochemical day was defined as continued 24 h OH exposure at a concentration of 1.5 × 10^6^ molecules per cm^3^.^60,61^

The acidity of the wood pellet burning aerosol was estimated using a procedure similar to that by Kodros *et al.*,^62^ using the aerosol thermodynamics model ISORROPIA-lite.^63^ ISORROPIA-lite is based on the ISORROPIA-II^64^ model but extended to account for the effects of water uptake from the organic aerosol that may exist, parameterized according to *κ*-Köhler theory.^65^ Inputs to the model include the relative humidity, temperature, the OA concentration, hygroscopicity parameter *κ* and density and the total (gas- and particle-phase) concentrations of ammonia/ammonium, sulfuric acid, sodium, calcium, potassium, magnesium, chloride/hydrochloric acid, and nitrate/nitric acid. A *κ* value equal to 0.15 was used.^66^ We assumed that the total nitric acid and hydrochloric acid concentrations were equal to the AMS-measured inorganic nitrate and chloride concentrations, respectively. This is a good assumption, given that the biomass burning aerosol pH is expected to be high enough so that most of the nitrate and chloride are in the aerosol phase.^62,67^ The potassium concentration was scaled as 15% of the PM nitrate.^68^ The concentrations of non-volatile cations such as sodium, calcium, and magnesium were assumed to be zero as their contribution to the sub-micrometer mode is considered to be negligible.^69^ For the submicron particles considered here and room temperature, the above methodology for pH estimation is expected to be reliable, albeit with the same limitations as when applied to ambient data. Pye *et al.*^69^ provide a thorough summary of the literature on the applicability of equilibrium models and associated errors for pH estimation.

## 3. Results and discussion

### 3.1 Characterization of fresh emissions

The initial OA concentration from the pellet stove emissions in the chamber ranged from approximately 40 to 200 μg m^−3^. The RH varied from low levels (about 10%; Exp. 1–3 and 7–9) to moderate (around 60%; Exp. 6) and high (up to 80%; Exp. 4 and 5). The average temperature of the experiments was 21 ± 1 °C (Table 1), a little higher than the typical average ambient temperature in Greece during winter periods which can be as high as 19 °C (*e.g.* ranging from 6–19 °C in Athens, Greece).^38^ These elevated temperatures along with high ozone concentrations in the atmosphere favor the formation of nitrate radicals. The relatively higher temperature during this set of experiments along with the ozone and NO_2_ additions aims to promote NO_3_ radical formation and create conditions for faster chemistry.

The time dependent AMS CE was determined to be on average 0.8 ± 0.2 (one standard deviation, SD) and the average OA density calculated based on the Kostenidou *et al.*^52^ algorithm was 1.21 ± 0.07 (mean ± SD) g cm^−3^. For comparison, the estimated OA density based on the measured O : C and hydrogen-to-carbon (H : C) elemental ratios as suggested by Kuwata *et al.*^70^ was on average 1.26 ± 0.02 (mean ± SD) g cm^−3^. Based on the results of the two methods, an average density of 1.25 g cm^−3^ was selected to characterize the fresh pellet emissions.

In all experiments, the fresh pellet stove emissions were dominated by OA, which represented 93 ± 4% of the measured PM_1_. Nitrate and OA had similar size distributions and mean diameters, indicating an internally mixed aerosol. With the exception of nitrate, the concentrations of the rest of the aerosol components measured using the AMS (sum of sulfate, ammonium and chloride) accounted for less than 3% of the aerosol mass (Table 1). Due to their low concentrations their corresponding AMS size distributions were quite noisy. The measured BC varied between 0.6 and 7 μg m^−3^, accounting for 1–8% of the measured PM_1._ This is in contrast to the study by Bertrand *et al.*,^5^ where BC was the dominant component (90%) of the fresh pellet emission. This difference is likely related to the operating conditions of the stove used in that study, as well as the choice of fuel which in the present study had high energy density (>4.6 kW h kg^−1^) with less than 10% moisture and a low percentage of ash (<0.7). The average organic aerosol to black carbon ratio (OA/BC) was 34 ± 24 which is higher than that reported by Heringa *et al.*^7^ (2.5 ± 0.9). A high OA/BC ratio usually indicates good combustion conditions. Our results are consistent with the measurements of McClure *et al.*^71^ who tested a number of biomass fuels and reported OA/BC ratios varying between 0.3 and 10.^5^ For example, for pine the OA/BC ranged between 7 and 143, while in our case the OA/BC ranged between 14 and 83.

Total nitrate (inorganic plus organic) accounted on average for 0.5–4% of the PM_1_ ranging between 0.4 and 2.5 μg m^−3^, while sulfate and chloride contributed equally 0.8%. Ammonium was less than 0.1% of the PM_1_ in all experiments. The organic nitrate fraction of total nitrate (see Section 2.2) was on average 31 ± 18% (Fig. S1†). The O : C of the fresh OA varied between 0.53 and 0.65 (Table 1) and is consistent with that in previous studies.^5,7^ The organic-mass-to-organic-carbon ratio (OM/OC) was on average 1.92 ± 0.04 (mean ± SD).

**Fig. 2:**
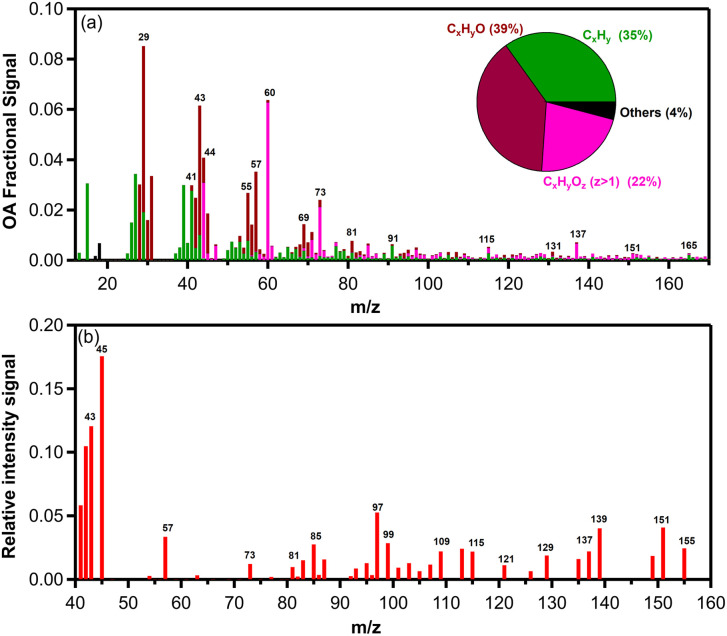
Average mass spectra of (a) organic aerosols and (b) gas-phase organics emitted by the pellet stove from AMS and PTR-MS, respectively. The high-resolution OA mass spectra for the main families and their average contribution are shown. All other families are presented in black colour.

The AMS mass spectrum of the fresh biomass burning OA (bbOA) exhibited moderate variability across the various experiments with the theta angle between pairs of experiments varying between 3 and 18° (Table S1†). The average high resolution (HR) fresh OA spectrum from all experiments is shown in Fig. 2. The spectrum is characterized by prominent peaks at *m*/*z* 29 (CHO^+^ and C_2_H_5_^+^), 39 (C_3_H_3_^+^), 43 (C_2_H_3_O^+^), 44 (CO_2_^+^ and C_2_H_4_O^+^), 55 (C_3_H_3_O^+^ and C_4_H_7_^+^), 57 (C_3_H_5_O^+^), 60 (C_2_H_4_O_2_^+^), 73 (C_3_H_5_O_2_^+^), 91 (C_7_H_7_^+^), 115 (C_9_H_7_^+^), 137 (C_8_H_9_O_2_^+^) and 151 (C_8_H_7_O_3_^+^). These fragments contributed 36 ± 2% of the total organic mass. The OA spectrum resembled the HR spectrum of primary pellet stove emissions reported in previous studies^5^ but with a stronger signal at *m*/*z* 29, 43, 44, 60, 73 and 137. The HR spectrum of OA mainly consisted of C_*x*_H_*y*_O^+^ (39%), followed by C_*x*_H_*y*_^+^ (35%) and C_*x*_H_*y*_O_*z*_^+^ (22%) with the rest of the families contributing 4% (Fig. 2a). The variability of the OA mass spectra of all fresh pellet emissions is shown in Fig. S1.†

While various VOCs are emitted by biomass burning, the PTR-MS measures only a limited VOC range (the ones that can be protonated). The measured VOC to NO_*x*_ ratios for the different experiments are shown in Table 1. The main gas-phase compounds and/or fragments of compounds measured using the PTR-MS in the environmental chamber after the injection period were phenolic compounds, furans and monoterpenes (Fig. 2b).

The major phenolic compounds detected were phenol (*m*/*z* 95), cresol (*m*/*z* 109), creosol (*m*/*z* 139), and 4-vinylguaiacol (*m*/*z* 151) which are commonly formed from thermal decomposition of lignin and are a significant component of biomass burning emissions.^32,72^ The *m*/*z* 151 could also be related to pinonaldehyde, but 4-vinylguaiacol was detected in previous studies of fire emissions.^72^

Furans are emitted into the atmosphere during biomass burning as products of the pyrolysis of cellulose.^73^ Furanoic emissions were dominated by furfural (*m*/*z* 97), which is considered a biomass burning marker^74^ and was also detected in pellet emissions.^14^ Monoterpenes (*m*/*z* 81 and *m*/*z* 137) detected with the PTR-MS were significant in all experiments. Other gas-phase compounds that were measured in the pellet emissions were: *m*/*z* 43 (alkyl fragments, propylene, acetic acid, acetone, and peroxyacetyl nitrate (PAN)), *m*/*z* 45 (acetaldehyde), *m*/*z* 57 (acrolein), *m*/*z* 73 (methyl ethyl ketone (MEK)), *m*/*z* 85 (ethyl vinyl ketone (EVK)), and *m*/*z* 87 (2-methyl-3-buten-2-ol (MBO)). Some of these VOCs (*m*/*z* 43, 45, 99, and 139) have been detected in pellet stove exhaust previously.^14,75^ Aromatic hydrocarbons such as benzene (*m*/*z* 79), toluene (*m*/*z* 93), xylenes/ethylbenzene (*m*/*z* 107), C10 aromatics (*m*/*z* 135), and C11 aromatics (*m*/*z* 149) were present in the emissions but were less than 1–2 ppb.

The modified combustion efficiency (MCE) is defined as the ratio of carbon emitted as carbon dioxide (CO_2_) to the total amount of carbon emitted as CO_2_ and CO and is a key metric of combustion.^76^ In general, a MCE value equal to one corresponds to complete combustion, while values between 0.9 and 1 indicate flaming conditions, and values of 0.85 and less indicate smoldering conditions.^77^ The arithmetic average MCE from the 9 experiments was 0.94 ± 0.02 (mean ± SD), with the lowest MCE values calculated for experiments 4–6 (Table 1).

In all experiments, the particle number distribution of the fresh emissions was unimodal. The calculated mean particle mobility diameter from the SMPS measurements during the emission period varied between 85 and 125 nm, with an average diameter of 106 nm and a modal width, *σ*, equal to 1.54. The variability in particle diameter is due to both small changes in the combustion conditions and coagulation of the fresh emissions. In previous studies, the number size distribution of pellet emissions was dominated by submicron particles with a maximum size in the range of around 150 nm.^78^

In two experiments the emissions were allowed to reside undisturbed in the dark chamber for 12 h. After particle wall-loss corrections were applied, no detectable changes in the mass, AMS spectrum and O : C ratio were observed, suggesting that low and semi-volatile vapor wall losses were not a dominant factor in our experiments.

### 3.2 Estimated volatility distributions of OA components

A TD was used during experiments 8 and 9 (dark reference experiments) in order to measure the volatility distribution of the fresh OA. Only the data of experiment 8 are presented here as both experiments resulted in similar conclusions. The OA spectrum and the O : C of Exp. 8 are quite similar with the rest of the experiments, while the *f*_44_/*f*_60_ was equal to 1.57, which is higher than that of the others (Table 1 and Fig. S1†).

The TD operated in a temperature range between 25 °C and 185 °C using several temperature steps. The centerline residence time of the aerosol in the TD was 16 s at 298 K. The OA mass fraction remaining (MFR) was calculated as the ratio of the organic mass concentration of a sample passing through the TD at time *t*_*i*_ over the average mass concentration of the bypass sample at times *t*_*i*−1_ and *t*_*i*+1_.

The thermodenuded OA was corrected for the AMS CE (average CE_TD_ = 0.9 ± 0.04) and for particle losses in the TD. The particle losses were measured as a function of temperature and particle size in an additional experiment using sodium chloride particles. The average loss fraction in the 0.1–1 μm size range for each temperature was used for the correction of the AMS results.^47^

**Fig. 3:**
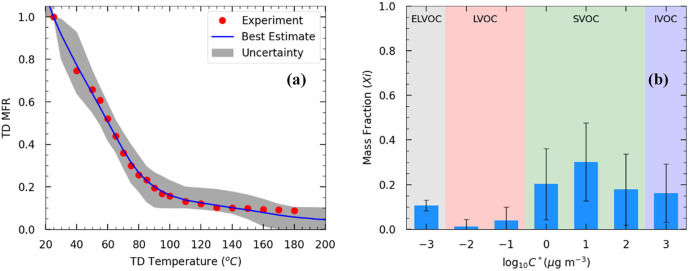
(a) Thermogram of the OA TD measurements of Exp. 8. Red dots represent the loss-corrected measurements. The black line represents the best estimate, and the gray area is the uncertainty range estimated using the model by Karnezi *et al.*^48^ (b) Estimated volatility distributions of the OA. The error bars represent the uncertainty calculated using the model. The ELVOCs in the 1 D-VBS framework are in the gray area, the LVOCs are in the red, the SVOCs are in the green, and the IVOCs are in the blue-shaded area.

The initial mass concentration in the TD was 70 μg m^−3^ and the mean volume diameter was 170 nm. The OA density (*ρ*_OA_) was equal to 1.25 g cm^−3^. Approximately 80% of the OA evaporated at 90 °C (Fig. 3a). At 180 °C, 9% of the initial OA remained in the particulate phase.

Applying the approach by Karnezi *et al.*,^48^ we determined the volatility distribution for the emitted OA by pellets. The model reproduced the measurements well (Fig. 3b). Our model results suggest that the pellet OA at 70 μg m^−3^ consisted of 11% extremely low-volatility compounds (ELVOCs), 5% low-volatility compounds (LVOCs), 68% semi-volatile compounds (SVOCs), and 16% intermediate-volatility compounds (IVOCs) (Fig. 3b). The estimated effective enthalpy of vaporization was equal to 77 ± 20 (mean ± SD) kJ mol^−1^, and the effective accommodation coefficient was 0.2 with an uncertainty range covering more than an order of magnitude.

### 3.3 Dark aging experiments

#### 3.3.1 Results of a typical dark oxidation experiment

**Fig. 4:**
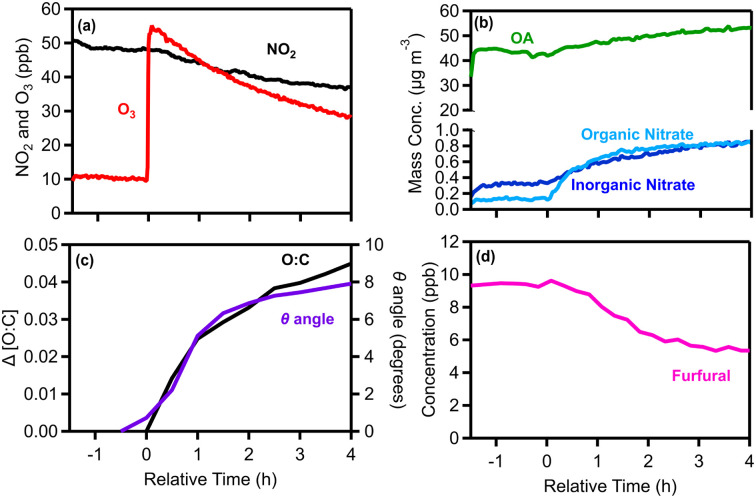
Measurements for experiment 1 (typical dark oxidation experiment) showing (a) NO_2_ and O_3_, (b) wall-loss corrected OA, particulate inorganic and organic nitrate, (c) the change in the O : C ratio and theta angle and (d) furfural; *m*/*z* 97.

During a typical experiment (Exp. 1), 50 ppb of NO_2_ were injected into the chamber at *t* = −2.5 h. Half an hour later the pellet emissions were added to the reactor (Fig. 4). The freshly emitted particles were composed mainly of OA (44 μg m^−3^; 97% of measured PM_1_). They also contained 0.6 μg m^−3^ of BC and less than 0.5 μg m^−3^ each of nitrate, sulfate, ammonium, and chloride. The BC in this experiment was the lowest in terms of mass concentration and percentage of the measured PM_1_ across all experiments (Table 1).

After leaving the pellet emissions for approximately 2 h in the dark, reactions were initiated with an injection of 55 ppb of O_3_ (*t* = 0) (Fig. 4a). Then the system was monitored for another 4 h for chemical processing and characterization of the aged aerosol and vapors. Following the O_3_ injection, formation of SOA and particulate organic nitrate was observed (Fig. 4b). After 4 h the OA increased by 11 μg m^−3^ (an increase of 21%), while the organic nitrate increased by 0.7 μg m^−3^ (Fig. 4b).

The degree of chemical processing of biomass burning OA (bbOA) was quantified using both the change in the O : C and the theta angle of its AMS spectrum compared with that of the fresh OA. Before time zero and the initiation of dark oxidation, both metrics were stable (change less than 3%), suggesting that the bbOA was well mixed and stable in the chamber (Fig. 4c). After 4 h of dark aging following the O_3_ injection, the O : C ratio increased by 0.05 (10% increase), while a small change in the OA AMS spectrum was observed (*θ* = 8°) (Fig. 4c), indicating evolution of the bbOA spectrum to some extent, but still maintaining similarity to the fresh bbOA.

During the same period the levels of several VOCs decreased. Furfural (*m*/*z* 97) displayed higher reduction (45%) (Fig. 4d), while phenolic compounds such as phenol (*m*/*z* 95) and cresol (*m*/*z* 109) both decreased by 13%. Monoterpenes (*m*/*z* 81 and *m*/*z* 137) were reduced by around 10% and xylenes/ethylbenzene (*m*/*z* 107) presented the most significant reduction (7%) amongst the aromatic hydrocarbons. We also observed a moderately increasing trend in hexenal (*m*/*z* 99; 10%), MEK (*m*/*z* 73; 6%) and toluene (*m*/*z* 93; 5%).

The estimated average OH concentration from the d-9 butanol decay was 1.4 × 10^6^ molecules per cm^3^. This is a relatively low level, but it is not negligible. We also calculated the production rate of NO_3_ based on the NO_2_ and O_3_ concentrations in the absence of VOCs. In this experiment, within the first hour the production rate of NO_3_ radicals increased from 1.2 to 6.5 ppb h^−1^ (Fig. S2†). To evaluate the relative contribution of reactions with nitrate radical, ozone and OH to the oxidation of pellet emissions, we calculated the average lifetime of the VOCs with the largest decreasing trends: furfural, phenol, cresol, creosol, and monoterpenes (assumed to be α-pinene for this calculation). We used the reaction rate constants reported in Table S2.† For OH, we used its concentrations in the first hour after time zero. For the NO_3_ radical concentration, we assumed an average concentration of 8 × 10^8^ molecules per cm^3^, as by Kodros *et al.*^62^ for a similar system.

For furfural, phenol and α-pinene, the average lifetimes for reactions with OH are greater than 6 hours (Table S3†). The corresponding lifetimes for reactions with NO_3_ are all less than half an hour for typical concentrations (0.14 h for phenol, 0.5 h for furfural, and 0.28 h for α-pinene), indicating that NO_3_ was the dominant oxidant of these VOCs in our experiments. Cresol and α-pinene have an average lifetime of more than a day and 1.3 h, respectively when reacting with ozone while the corresponding furfural reaction is very slow.^79,80^

Creosol and cresol display an average lifetime, for reaction with NO_3_, close to that of OH (2 h for creosol and 4 h for cresol), implying that their oxidation by the two oxidants was competitive. So, together with the NO_3_ reactions there were also some reactions with OH taking place, but their overall extent was secondary.

#### 3.3.2 Results of other experiments

In all dark-aging experiments (experiments 1–6) the O : C remained roughly constant prior to oxidation (changing by less than 3%). In experiments 1 and 5, we used lower NO_2_ and O_3_ concentrations (nearly a factor of 2) than in the other experiments (Table 1), that resulted in lower SOA levels and smaller changes in the OA AMS spectrum. The dark aging experiments have been separated into two categories, low RH (∼10%) (Exp. 1–3) and higher RH (>60%) experiments (Exp. 4–6). The average concentration of OH was 1.8 × 10^6^ molecules per cm^3^ (ranging between 1.4 and 2.1 × 10^6^ molecules per cm^3^).

**Fig. 5:**
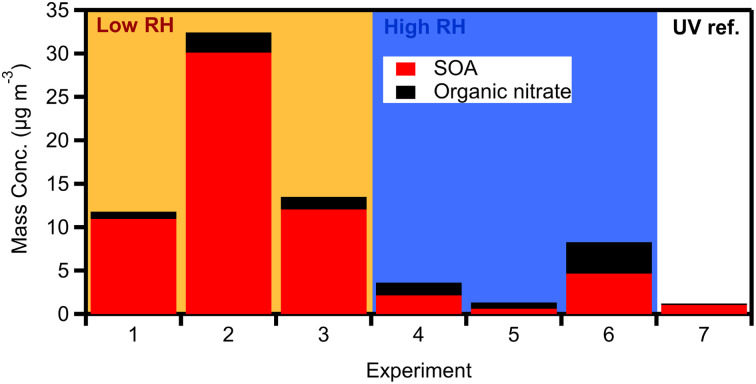
Produced OA (red bars) and organic nitrate (black bars) for the dark aging (1–6) and UV (7) experiments. The values correspond to diluted concentrations in the chamber.

**Fig. 6:**
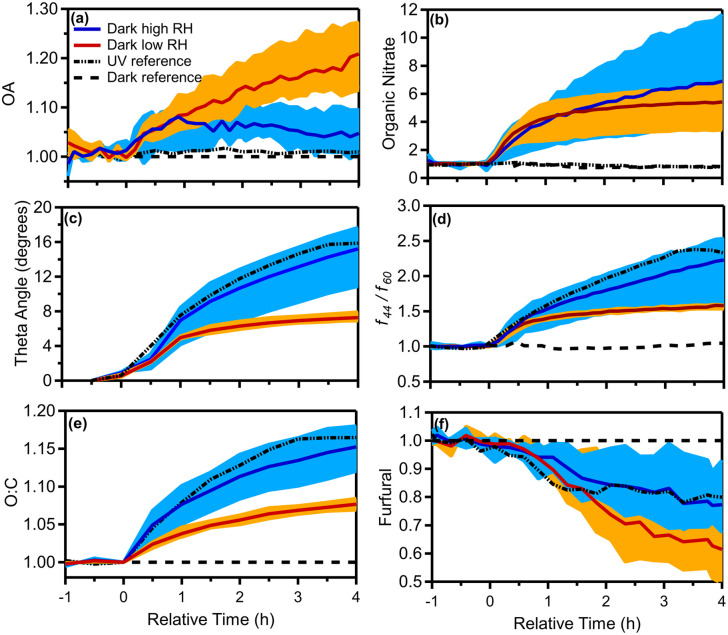
Enhancement (normalized to initial values of 1) in (a) OA, (b) organic nitrate, (c) change in theta angle in degrees, (d) *f*_44_/*f*_60_, (e) O : C ratio, and (f) furfural (*m*/*z* 97) for the dark aging experiments under dry (experiments 1 to 3) and high RH (experiments 4 to 6) conditions. The shaded blue (dark RH experiments) and orange (dark dry experiments) regions correspond to the variability across all experiments due to differences in injected NO_2_ and O_3_ concentrations, while the solid blue and red lines are the median across the RH and dry experiments, respectively, and the dashed black lines correspond to UV (experiment 7) and dark reference (experiment 8) experiments.

Fig. 5 shows the produced OA (including organic nitrate) in all dark aging experiments. The formed SOA ranged between 0.6 and 30 μg m^−3^, increasing the OA levels by 1–28% (Fig. 6a) after a few hours of exposure to NO_3_ radicals in the chamber. For the low RH experiments, the formed SOA after 4 h of oxidation ranged from 12–28% of the initial OA (11–30 μg m^−3^ increase). Dark experiments under humid conditions showed a 1–10% increase in OA, forming clearly less SOA (0.6–5 μg m^−3^) in comparison to low RH conditions (Fig. 6a). This indicates that dryer nights in “rich” biomass burning urban haze episodes may have a lot more SOA than under humid conditions.

After the injection of NO_2_ and O_3_, both organic and inorganic nitrates increased. Organic nitrate increased in all experiments by 200–600%, while in the case of experiment 5 it increased up to 1000%, reaching values up to 1 μg m^−3^ (Fig. 6b). For the low RH set of dark aging experiments, the mass concentration of organic nitrate increased by 300–600% (corresponding to 0.7–2 μg m^−3^), while for the higher RH cases the produced secondary organic nitrate after 4 h ranged from 400 to 1000% (an increase of 0.9 to 1.6 μg m^−3^; Fig. 6b). Inorganic nitrate increased by 30–230% (0.6–2.7 μg m^−3^) in all experiments, with higher RH cases presenting an average increase of 80% and lower RH cases 170% (Fig. S3†). The enhancements of organic and inorganic nitrate aerosols are typical of dark aging due to the presence of NO_3_ radicals.

There was a minor increase in OA density (ranging from 3 to 7%) during the aging period. This increase was a bit higher in the case of higher RH experiments (4 to 7%; absolute change of 0.04 to 0.09 g cm^−3^) in comparison to the 3 to 4% (absolute change of 0.03 to 0.05 g cm^−3^) difference in low RH cases.

The theta angle between the fresh and aged OA spectra increased in all dark-aging experiments by 7–18°, in the 4 h of oxidation (Fig. 6c). The changes in the OA mass spectrum were smaller under low RH conditions. The theta angle changed around 6° in the first hour of dark oxidation and only 1–2 additional degrees during the next 3 hours.

For experiments 4–6 (elevated RH) the increase in the theta angle was almost twice (*θ* = 10–18°) at the end of the experiment (Fig. 6c), in comparison to low RH cases. Given the relatively low SOA formation at high RH, heterogeneous reactions in this system that oxidize the original OA appear to be important and deserve additional investigation. The theta angle between the aged spectra at *t* = 4 h for all experiments was also calculated (Table S4†). The theta angle between the aged spectra at *t* = 4 h for all experiments ranged between 4 and 8° in the case of low RH conditions (Exp. 1–3) and up to 20° for high RH cases (Table S4†). The average spectra for the dry and high RH cases, along with the corresponding deviations are shown in Fig. S5.†

**Fig. 7:**
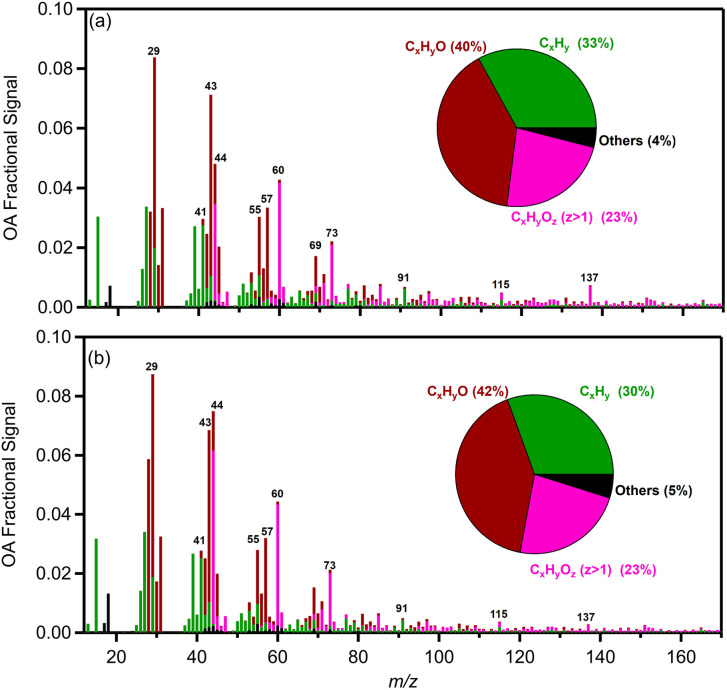
Average high-resolution mass spectra for the organic mass spectra of aged pellets for (a) low and (b) high RH cases. The average contribution of the main components is also shown. All other families are presented in black colour.

For the high RH experiments in which the aged OA spectrum changed considerably, the levels of the C_*x*_H_*y*_^+^ family displayed a noticeable decrease (5%) compared to the fresh bbOA (Fig. 7). The fraction of the C_*x*_H_*y*_O^+^ family increased slightly (3%), while the C_*x*_H_*y*_O_*z*_^+^ and C_*x*_H_*y*_N^+^ families' contributions did not change significantly (change of 1%). The main differences between the fresh bbOA spectrum and that of elevated RH cases were found for *m*/*z* 28 and 44 which are more pronounced in the aged bbOA spectrum. The *m*/*z* 60 and 137 were lower in the aged bbOA spectra (Fig. 7b).

To quantify the evolution of OA composition, we examined the ratio of the OA mass signal at *m*/*z* 44 to *m*/*z* 60 (*f*_44_/*f*_60_) and the O : C. In the dark aging experiments after the injection of NO_2_ and O_3_ (precursors to the NO_3_ radical), an enhancement in *f*_44_/*f*_60_ by a factor of 1.5 to 2.5 was observed under both dry and high RH conditions (Fig. 6d). The dark aging of bbOA seems to be sensitive to RH suggesting a sensitivity of the aging process to water vapor and/or aerosol liquid water content. The enhancement for the low RH experiments was similar in all experiments, reaching its maximum of 1.5 after 30 min and increasing slowly during the rest of the experiments. The corresponding enhancement for high RH experiments increased throughout the experiment and was quite variable (1.5 to 2.5) (Table 2).

**Table 2:** Concentration of PM_1_ components (in μg m^−3^) and VOCs (in ppb) for low and high RH for fresh emissions and after dark aging periods. All concentrations are those measured in the chamber after dilution

Species	Low RH		High RH	
	Fresh emissions	Dark aging	Fresh emissions	Dark aging
	**μg m^−^** ^**3**^		**μg m^−^** ^**3**^	
Ammonium	0.07 ± 0.06	0.05 ± 0.05	0.03 ± 0.03	0.05 ± 0.02
Sulfate	0.22 ± 0.12	0.23 ± 0.10	0.70 ± 0.94	0.78 ± 1.04
Organics	113.00 ± 82.83	130.20 ± 92.45	66.00 ± 10.58	66.89 ± 10.78
Nitrate	1.10 ± 0.82	3.72 ± 2.41	2.90 ± 1.85	5.22 ± 2.00
Chloride	0.23 ± 0.17	0.12 ± 0.12	0.47 ± 0.65	0.08 ± 0.09
BC	4.07 ± 3.20	4.02 ± 3.12	4.23 ± 1.25	3.87 ± 1.22

	**ppb**		**ppb**	
NO_2_	80.00 ± 26.46	46.15 ± 20.47	80.00 ± 36.06	37.97 ± 11.55
NO	7.32 ± 2.60	3.02 ± 1.86	6.62 ± 2.49	3.26 ± 1.51
O_3_	85.00 ± 25.98	36.32 ± 7.11	78.33 ± 29.30	31.53 ± 9.29
*m*/*z* 42 (acetonitrile)	16.95 ± 9.28	16.62 ± 9.41	10.08 ± 4.23	10.06 ± 3.90
*m*/*z* 57 (acrolein)	6.76 ± 2.28	6.52 ± 2.08	5.87 ± 0.25	5.95 ± 0.20
*m*/*z* 73 (MEK)	4.05 ± 0.71	4.22 ± 0.74	3.33 ± 0.43	3.51 ± 0.17
*m*/*z* 81 (monoterpenes/hexanal)	3.65 ± 0.48	2.98 ± 0.23	3.09 ± 0.12	2.69 ± 0.06
*m*/*z* 93 (toluene)	1.07 ± 0.46	1.15 ± 0.35	1.16 ± 0.21	1.29 ± 0.03
*m*/*z* 95 (phenol)	4.98 ± 0.67	3.70 ± 0.31	4.50 ± 0.28	3.72 ± 0.19
*m*/*z* 97 (furfural)	9.87 ± 3.54	5.93 ± 1.09	7.69 ± 0.28	5.97 ± 0.19
*m*/*z* 99 (hexenal)	6.86 ± 1.80	7.36 ± 1.54	5.27 ± 0.23	5.69 ± 0.12
*m*/*z* 107 (xylenes)	1.57 ± 0.55	1.36 ± 0.57	1.30 ± 0.07	1.38 ± 0.19
*m*/*z* 109 (cresol)	8.58 ± 1.29	6.66 ± 0.45	9.29 ± 0.32	8.44 ± 0.56
*m*/*z* 115 (heptanal)	7.19 ± 1.43	6.67 ± 0.60	5.46 ± 0.23	5.72 ± 0.34
*m*/*z* 135 (C10 aromatics)	2.15 ± 0.60	1.68 ± 0.34	2.38 ± 0.55	2.60 ± 0.32
*m*/*z* 137 (monoterpenes)	10.69 ± 1.21	9.18 ± 0.76	9.23 ± 0.17	9.13 ± 0.37
*m*/*z* 139 (creosol, methyl-guaiacol)	13.53 ± 2.28	11.18 ± 0.59	11.40 ± 0.27	9.97 ± 0.24
*m*/*z* 149 (C11 aromatics)	6.52 ± 0.98	6.91 ± 0.49	2.35 ± 0.50	2.49 ± 0.32
*m*/*z* 151 (4-vinylguaiacol)	15.74 ± 2.44	13.25 ± 1.03	13.20 ± 0.30	13.02 ± 0.60

The O : C ratio increased in all experiments by 7–18% in 4 h (an absolute increase of 0.04–0.12). For experiment 5, which was performed at elevated RH (80%) but a relatively low concentration of NO_2_ and O_3_ (40 and 45 ppb, respectively), the corresponding increase in O : C (12%) was the lowest across the three high RH experiments. For the low RH experiments, the average O : C at 4 h was 0.59 ± 0.01 (mean ± SD), while for the higher RH cases, the corresponding value was 0.70 ± 0.06. The Van Krevelen (VK) diagram^81^ presents the relation between the H : C and O : C ratios. The OA components for the pellet-burning experiments shown here mostly fall outside the VK-triangle region (Fig. S6†). For low RH experiments the enhancement in aging metrics is accompanied by decreasing concentrations in most of the measured VOCs. The highest decrease was detected for furfural (*m*/*z* 97; 40 ± 14%), phenol (*m*/*z* 95; 26 ± 8%), cresol (*m*/*z* 109; 22 ± 3%), monoterpenes/hexanal (*m*/*z* 81; 20 ± 7%), creosol (*m*/*z* 139; 20 ± 10%) and monoterpenes (*m*/*z* 137; 14 ± 6%) (Fig. S4†). This is in agreement with previous studies as biomass-burning-related VOCs, such as furfural and monoterpenes (C_10_H_16_), can react with NO_3_ radicals, resulting in nighttime SOA production.^73,82,83^ Palm *et al.*^84^ suggested that nighttime NO_3_ oxidation of phenolic compounds in bbOA plumes leads to nitrophenolics, which considerably contribute to SOA production. A small increase was detected for hexenal (*m*/*z* 99; 8 ± 6%), MEK (*m*/*z* 73; 5 ± 2%) and toluene (*m*/*z* 93; 8 ± 2%).

In the high RH set of experiments, a reduction in the concentration for the majority of VOCs (Fig. S4†) was detected, but the decrease was lower than that during low RH experiments (in some cases even half). Furfural for example decreased to 22 ± 12%, followed by a decrease in phenol (17 ± 4%), monoterpenes (*m*/*z* 81; 13 ± 4%), creosol (13 ± 1%), and cresol (10 ± 3%). At the same time certain VOCs (*e.g.*, toluene, xylene, and hexenal) appeared to increase a little, but most of these changes were within experimental error. A fraction of these changes may also be due to the production of the corresponding compounds from reactions of larger molecules.

In all cases O_3_ decreased to approximately 30 ppb (a decrease of 54 ± 7%) in the 4 h of oxidation, with the lowest decrease observed for the low oxidant cases. The NO_2_ followed the same trend as ozone decreasing to 45 ± 20%.

#### 3.3.3 Daytime oxidation of pellet emissions

In experiment 7 we initiated oxidation (at time zero) by turning on the UV lights to simulate daytime (OH radical dominated) oxidation, without adding nitrous acid or NO_2_/O_3_. The initial O_3_ concentration (prior to turning UV lights on) was 10 ppb and it reached 30 ppb in 180 min (Fig. S7†). In contrast, Reyes *et al.*^29^ found no increase in O_3_ with UV lights in their similar experiment.

At the same time SOA formation of around 2 μg m^−3^ (2% increase in OA) was observed (Fig. S7a†). This is comparable to the formed SOA in dark experiment 5 and consistent with the low or no SOA mass formed by pellet emissions for the flaming phase under UV exposure in previous studies.^7,29^ This may be explained by the good burning conditions for the pellet stove but it may also be due to the specific feed used. Organic and inorganic nitrates did not increase under these daytime conditions (Fig. S7a†).

The O : C in this experiment increased to 0.63 (0.09 absolute increase or a 17% increase; Fig. S7c†) comparable to that of the high RH experiments under dark conditions. The aged OA spectrum changed in comparison to that of fresh emissions by a theta angle of 16°, similar to the 10–18° in the high RH dark-aging experiments. The fraction of *m*/*z* 60 (due to C_2_H_4_O_2_^+^, characteristic of levoglucosan which is used as a molecular marker in biomass burning) was *f*_60_ = 0.048 in the fresh bbOA spectrum and decreased to 0.035 in the corresponding aged spectrum.

After the 3 h of UV illumination the lights were turned off and the system remained dark for another two hours to monitor any change in the OA spectrum and O : C ratio. Both the *θ* angle and O : C did not change further.

The levels of most VOCs decreased after the UV lights were switched on. The highest decreases were observed for *m*/*z* 73, 81, 85, 87, 95, 97, 109, 139 and 151 (pinonaldehyde/4-vinylguaiacol) (Fig. S4†). The highest decrease (27%) was detected for *m*/*z* 139 (creosol and methyl-guaiacol) followed by MEK and furfural (*m*/*z* 73 and 97; −21% each) (Fig. S7d†). A slight increase (6–9%) was detected in *m*/*z* 31 (formaldehyde), 42 (acetonitrile), 47 (formic acid), 59 (acetone), 61 (acetic acid), 71 (methyl vinyl ketone (MVK) or methacrolein (MACR)) and 79 (benzene).

#### 3.3.4 Dark reference experiments (no oxidants)

We also performed two additional dark reference experiments (Exp. 8 and 9) without addition of oxidants such as NO_2_ or O_3_. In experiment 8, the pellet emissions remained undisturbed in the chamber for approximately 12 hours. The high-resolution OA mass spectrum remained constant during that period (final *θ* = 3.1°). The O : C initially was 0.58 and after 12 h changed slightly to 0.60 (5.3% increase). The mass spectrum of experiment 9 after 4 h remained almost the same (*θ* = 5°). The corresponding O : C for time zero was 0.64 and increased to 0.67 after 4 h (4.7% increase). These suggest that the chemical interactions between the bbOA and the dark atmospheric simulation chamber (especially its walls) are minor for periods much longer than the duration of our experiments.

### 3.4 Aerosol acidity

We simulated the thermodynamic state of the pellet emissions (see Section 2.2), in order to evaluate the aerosol acidity of the fresh and aged emissions. The OA hygroscopicity parameter *κ* was assumed to be equal to 0.15. The OA density for the fresh emissions was equal to 1.25 g cm^−3^ (see Section 3.1), while for the aged OA the density ranged between 1.28 and 1.35 g cm^−3^, depending on the experiment. Sensitivity tests with or without including OA and potassium were performed (Fig. S8†) and different values (between 0.1 and 0.2) of the OA hygroscopicity parameter *κ* were used, but little change in estimated pH was found.

**Fig. 8:**
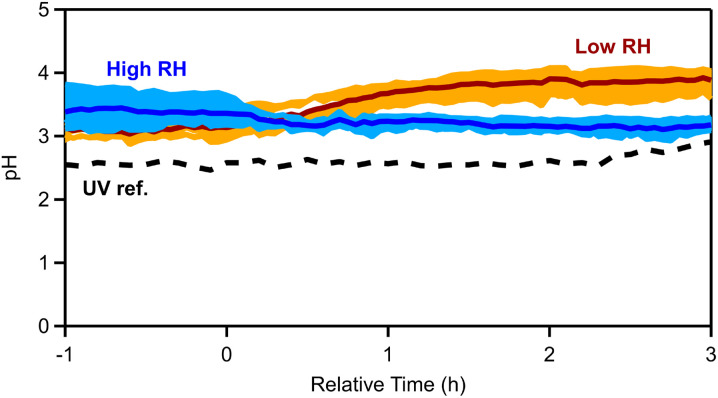
Estimated aerosol pH for all dark experiments (light blue shaded regions correspond to the variability across the high RH, while orange corresponds to the dry experiments, with solid blue and red lines being the median across the RH and dry experiments, respectively) and dashed black lines correspond to the UV reference experiment.

For the fresh emissions an average pH of 3.2 ± 0.4 was calculated for all dark experiments. By splitting the experiments into low and high RH sets, the pH for the low RH conditions was estimated to be equal to 3.1 ± 0.1, while for cases with elevated RH the pH was 3.4 ± 0.3 (Fig. 8). This is expected given that higher RH is associated with higher liquid water contents that tend to dilute aerosol acidity.^69^ The estimated pH changed after the ozone injection. For low RH cases, the pH increased to 3.9 ± 0.2 (a 0.8-unit increase; Table S8†) at *t* = 4 h, while at high RH the pH decreased to 3.1 ± 0.1 (0.3-unit decrease). In the reference experiment with UV lights (Exp. 7), the pH of the aerosol increased from 2.5 to 2.8 (0.3-unit increase).

### 3.5 Nighttime SOA formation: wood *vs.* pellets

Our results can first be compared to those of a similar study^62^ of biomass burning woodstove emissions in the same facilities under similar dark aging conditions using NO_2_ and O_3_. In the current study, OA was the dominant fresh aerosol compound (>90%) in all experiments. For the wood stove experiments, organics were responsible for 70% of the measured PM_1_, and BC accounted on average for 26%. The discrepancy in BC emissions indicates the better combustion performance of the pellet stove compared to the logwood one. The total nitrate in both studies was on average 1–2%, with organic nitrate being responsible for 33–56% of PM nitrate in the case of wood log burning and 4–54% for pellets. Sulfate and ammonium were responsible for less than 1% of the measured PM_1_ in both studies.

The average OA spectra of fresh bbOA emissions from wood and pellet combustion had a theta angle of 20° (Fig. S9†) implying some similarity, but also significant differences. The main differences between the two spectra were found for the signals at *m*/*z* 29, 57, 60, 73, 77 (C_6_H_5_^+^), 124 (C_6_H_4_O_3_^+^), 137 and 167 (C_9_H_11_O_3_^+^). The peaks at *m*/*z* 29, 60 and 73 were much higher in the pellet spectra (almost three times in the case of *m*/*z* 60). The *m*/*z* 60 and 73 peaks are related to sugar anhydrides such as levoglucosan, mannosan and galactosan, which are produced during burning of biomass containing cellulose and hemicellulose and are considered tracers of bbOA.^85,86^ Previous studies have reported the percentages of *m*/*z* 60 for biomass burning to be in the range of 0.6% and 4.1% (ref. 87) which is consistent with the fresh olive bbOA (around 2% (ref. 33)). In the case of pellets the corresponding percentage is much higher and around 6%, out of the suggested range. This high peak at *m*/*z* 60 may be a possible tracer for pellet-related bbOA emissions. Other marker ions for biomass burning lignin-related monomers are the peaks at *m*/*z* 137 and 167,^88^ with the latter being visible only in the wood and not in the pellet spectrum.

The fresh pellet bbOA spectrum can also be compared to the corresponding bbOA spectra found in the AMS database (High-Resolution AMS Spectral Database)^89^ and other previous studies. The pellet spectrum was relatively similar (*θ* less than 20°) to the bbOA factors from campaigns in Fresno, Athens and Patras^38,90^ and shared some similarities with some of the spectra obtained during FLAME-I (Fire Lab at Missoula Experiment, Phase 1) such as the burning of Alaska core Tundra duff (*θ* = 22°), southern pine needles and mixed wood (both with *θ* of 25°). For the rest of the database spectra, the theta angle values exceeded 25° (Fig. S10†) reaching up to 50°. The angles with the smoldering bbOA spectra (not shown) exceeded 50°.

The average O : C or the fresh pellet bbOA was high and around 0.6. The corresponding O : C of fresh bbOA from logwood of Kodros *et al.*^62^ was in the range of 0.3–0.4, close to that in ambient studies, which usually report O : C values less than 0.4 for fresh bbOA emissions^91^ and higher than 0.7 for aged bbOA.^92^ The high O : C for pellets is associated with the high signal in *m*/*z* 60 (C_2_H_4_O_2_^+^) and is not due to *m*/*z* 44 (*f*_44_ was close to 0.04).

**Fig. 9:**
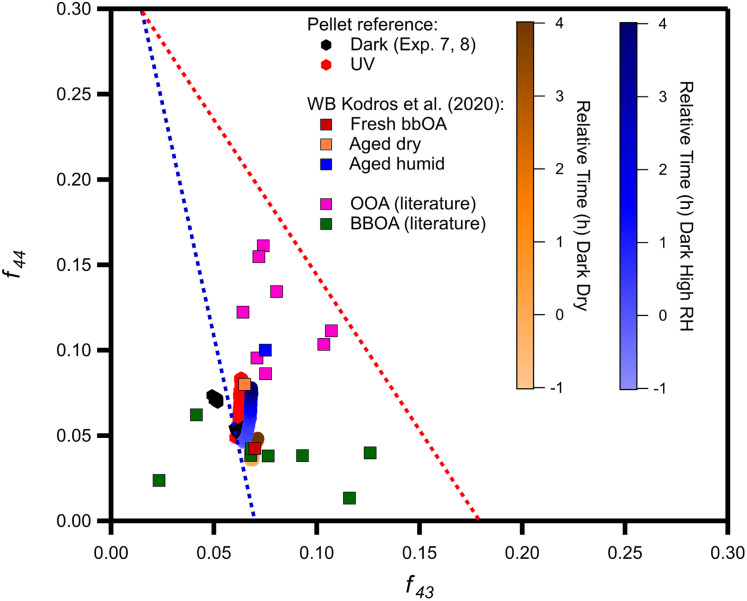
The oxidation pathway of the average dark dry (experiments 1–3; orange circles), average dark humid (experiments 4–6; blue circles), reference (Exp. 7 and 8; black) and UV (Exp. 9; red) experiments compared to ambient observations of OOA (pink squares), and bbOA (green squares) factors (ESI Table S6†).

The triangle plot in Fig. 9 displays the evolution of pellet bbOA during nighttime and daytime aging in our experiments. During the daytime the OA changes chemically and after 4 h it approaches in this space the aged under dry conditions bbOA spectrum of Kodros *et al.*^62^ and also some of the OOA factors found in winter periods. For dark aging the role of RH is evident. Under dry conditions, the aged bbOA remains in the same region as the fresh OA (Fig. 9), despite the fact that production of SOA is observed. In contrast, at higher RH the OA changes and moves closer to the area where wintertime OOA factors reside.

Dark oxidation for log wood burning emissions led to a significant enhancement in OA mass (up to 80%),^62^ which was much more than the increase reported here for pellets (up to 30%). The same picture applies also to the absolute change in the O : C. In this study the change in O : C varied between 0.04 and 0.12 (7–21% increase), while for the wood burning experiments the corresponding change was 0.09–0.23 (20–60% increase). This might be related to the higher initial O : C of the pellet OA. The final O : C after dark aging for both sources was close to 0.7, indicating that this might be the upper limit of oxidation under these conditions. In both studies, the experiments with the lowest increase in the O : C were those with the lowest levels of oxidants. Secondary nitrate aerosol was produced in all pellet and wood dark-aging experiments, with organic nitrate presenting a higher increase in both cases.

The dark aged bbOA spectra from pellets and wood log combustion were quite different. For low RH (10%) the spectra had a theta angle of 35° (Fig. S11a†) while for high RH it was a bit lower than 30° (Fig. S11b†). The high theta angles suggest that the aged OA spectra from the two sources can be potentially separated in ambient datasets by positive matrix factorization analysis or other source-apportionment techniques. The main differences between logs and pellets are found in AMS *m*/*z* 28 and 44, which are higher in the case of logwood emissions, and *m*/*z* 57, 60 and 73 which are higher in the aged pellet OA spectrum (Fig. S11†).

Phenol (*m*/*z* 95) was the major VOC consumed in the wood stove experiments while furfural (*m*/*z* 97) dominated for the pellets. Hexanal (*m*/*z* 83) was the major product detected in the wood dark aging experiments, while here it was mainly MEK (*m*/*z* 73) and hexenal (*m*/*z* 99).

The fresh pellet aerosol pH was 3.2, while Kodros *et al.*^62^ estimated a value of 2.8 for the wood bbOA pH. In the work by Kodros *et al.*,^62^ the pH of the aerosol remained constant and did not change significantly following dark oxidation. In contrast, in our study, the RH seems to play a major role, as for humid conditions the pH decreased by 0.3 units following dark oxidation (9% decrease) and increased by 0.8 units (26%) for low RH cases.

## 4. Conclusions

Previous research on the atmospheric fate of biomass burning emissions has focused on their photochemical aging, while nighttime aging processes have received relatively little attention. Our study indicates that significant SOA formation can take place in a few hours as pellet emissions react with NO_3_ radicals and other oxidants during the night. SOA production was higher under low RH conditions. For elevated RH (60–80%) little SOA (1–10% of the initial OA) was produced. Increases in the concentrations of oxidants led, as expected, to higher nighttime SOA production. The exposure of the pellet emissions to UV resulted in only a 2% increase in OA mass.

In all dark aging experiments furfural (*m*/*z* 97) was the measured VOC species that decreased the most, followed by monoterpenes/hexanal (*m*/*z* 81), phenol (*m*/*z* 95), cresol (*m*/*z* 109), monoterpenes (*m*/*z* 137) and creosol (*m*/*z* 139). These VOCs are expected to be the major contributors to the observed SOA formation.

Despite the relatively high SOA production at low RH, the bbOA AMS spectrum changed little and its O : C increased by only 7–8%. In contrast, at high RH even if there was little additional SOA formed the OA mass spectrum changed significantly and the aerosol became more oxidized. This finding suggests the importance of heterogeneous reactions that can change the composition of the bbOA without necessarily increasing its mass concentration significantly. Secondary nitrate aerosol was produced in all dark-aging experiments, with organic nitrate contributing on average 40% to total PM_1_ nitrate.

The fresh PM_1_ pellet emissions were dominated by organics (more than 90%), followed by BC and nitrate. The MCE found for the fresh bbOA (higher than 0.92) indicated flaming conditions, demonstrating good operation of the pellet stove and, thus, relatively efficient combustion conditions. The average fresh pellet bbOA spectrum resembles to some extent that of the one emitted by logs, with *m*/*z* at 57, 60, 73 and 167 presenting the main differences between the two spectra. The fresh bbOA at 70 μg m^−3^ consisted of extremely low-volatility (11%), low-volatility (5%), semi-volatile (68%) and intermediate-volatility (16%) compounds. Its estimated effective enthalpy of vaporization was equal to 77 ± 20 kJ mol^−1^. The fresh pellet bbOA displayed an average O : C ratio close to 0.6, which is associated with the high signal of *m*/*z* 60 (C_2_H_4_O_2_^+^).

Our results indicate significant differences in the aging of pellet and wood log burning emissions. This suggests that the corresponding emissions may need to be treated differently in chemical transport models that have so far lumped them together as residential biomass burning.

## Author contributions

A. N. and S. N. P. contributed to conceptualization; K. F., J. K. K., M. P., S. S. and M. M. contributed to investigation; K. F., J. K. K., S. J., and P. U. contributed to formal analysis; K. F. wrote the original draft; all authors contributed to review and editing.

## Conflicts of interest

The authors declare that are no conflicts to declare.

## Supplementary Material

EA-003-D3EA00070B-s001

## References

[cit1] Pandis S. N., Skyllakou K., Florou K., Kostenidou E., Kaltsonoudis C., Hasa E., Presto A. A. (2016). Urban particulate matter pollution: a tale of five cities. Faraday Discuss..

[cit2] Cristina Calderón E. and Colla M., Bioenergy Europe Statistical Report, Pellet Report, 2019, https://epc.bioenergyeurope.org/wp-content/uploads/2020/02/SR19_Pellet_final-web-1.pdf

[cit3] Obaidullah M. and De Ruyck J., in Renewable Energy - Technologies and Applications, IntechOpen, 2021

[cit4] Bäfver L. S., Leckner B., Tullin C., Berntsen M. (2011). Particle emissions from pellets stoves and modern and old-type wood stoves. Biomass Bioenergy.

[cit5] Bertrand A., Stefenelli G., Bruns E. A., Pieber S. M., Temime-Roussel B., Slowik J. G., Prévôt A. S. H., Wortham H., El Haddad I., Marchand N. (2017). Primary emissions and secondary aerosol production potential from woodstoves for residential heating: influence of the stove technology and combustion efficiency. Atmos. Environ..

[cit6] Corbin J. C., Keller A., Lohmann U., Burtscher H., Sierau B., Mensah A. A. (2015). Organic emissions from a wood stove and a pellet stove before and after simulated atmospheric aging. Aerosol Sci. Technol..

[cit7] Heringa M. F., DeCarlo P. F., Chirico R., Tritscher T., Dommen J., Weingartner E., Richter R., Wehrle G., Prévôt a. S. H., Baltensperger U. (2011). Investigations of primary and secondary particulate matter of different wood combustion appliances with a high-resolution time-of-flight aerosol mass spectrometer. Atmos. Chem. Phys..

[cit8] Ozgen S., Caserini S., Galante S., Giugliano M., Angelino E., Marongiu A., Hugony F., Migliavacca G., Morreale C. (2014). Emission factors from small scale appliances burning wood and pellets. Atmos. Environ..

[cit9] Reda A. A., Czech H., Schnelle-Kreis J., Sippula O., Orasche J., Weggler B., Abbaszade G., Arteaga-Salas J. M., Kortelainen M., Tissari J., Jokiniemi J., Streibel T., Zimmermann R. (2015). Analysis of gas-phase carbonyl compounds in emissions from modern wood combustion appliances: influence of wood type and combustion appliance. Energy Fuels.

[cit10] Akagi S. K., Yokelson R. J., Wiedinmyer C., Alvarado M. J., Reid J. S., Karl T., Crounse J. D., Wennberg P. O. (2011). Emission factors for open and domestic biomass burning for use in atmospheric models. Atmos. Chem. Phys..

[cit11] Bruns E. A., Krapf M., Orasche J., Huang Y., Zimmermann R., Drinovec L., Močnik G., El-Haddad I., Slowik J. G., Dommen J., Baltensperger U., Prévôt A. S. H. (2015). Characterization of primary and secondary wood combustion products generated under different burner loads. Atmos. Chem. Phys..

[cit12] Alfarra M. R., Prevot A. S. H., Szidat S., Sandradewi J., Weimer S., a Lanz V., Schreiber D., Mohr M., Baltensperger U. (2007). Identification of the mass spectral signature of organic aerosols from wood burning emissions. Environ. Sci. Technol..

[cit13] Fuller G. W., Tremper A. H., Baker T. D., Yttri K. E., Butterfield D. (2014). Contribution of wood burning to PM10 in London. Atmos. Environ..

[cit14] Czech H., Pieber S. M., Tiitta P., Sippula O., Kortelainen M., Lamberg H., Grigonyte J., Streibel T., Prévôt A. S. H., Jokiniemi J., Zimmermann R. (2017). Time-resolved analysis of primary volatile emissions and secondary aerosol formation potential from a small-scale pellet boiler. Atmos. Environ..

[cit15] Lamberg H., Nuutinen K., Tissari J., Ruusunen J., Yli-Pirilä P., Sippula O., Tapanainen M., Jalava P., Makkonen U., Teinilä K., Saarnio K., Hillamo R., Hirvonen M.-R., Jokiniemi J. (2011). Physicochemical characterization of fine particles from small-scale wood combustion. Atmos. Environ..

[cit16] Kinsey J. S., Touati A., Yelverton T. L. B., Aurell J., Cho S. H., Linak W. P., Gullett B. K. (2012). Emissions characterization of residential wood-fired hydronic heater technologies. Atmos. Environ..

[cit17] Johansson L. S., Leckner B., Gustavsson L., Cooper D., Tullin C., Potter A. (2004). Emission characteristics of modern and old-type residential boilers fired with wood logs and wood pellets. Atmos. Environ..

[cit18] Orasche J., Seidel T., Hartmann H., Schnelle-Kreis J., Chow J. C., Ruppert H., Zimmermann R. (2012). Comparison of emissions from wood combustion. Part 1: emission factors and characteristics from different small-scale residential heating appliances considering particulate matter and polycyclic aromatic hydrocarbon (PAH)-related toxicological potential of particle-bound organic species. Energy Fuels.

[cit19] Boman C., Pettersson E., Westerholm R., Boström D., Nordin A. (2011). Stove performance and emission characteristics in residential wood log and pellet combustion, part 1: pellet stoves. Energy Fuels.

[cit20] Gilles Gauthier I. A. , Bioenergy Europe Statistical Report, Pellet Report 2020, 2020, https://bioenergyeurope.org/article/268-pellet.html, cited 2023 January 30

[cit21] Sippula O., Hytönen K., Tissari J., Raunemaa T., Jokiniemi J. (2007). Effect of Wood Fuel on the Emissions from a Top-Feed Pellet Stove. Energy Fuels.

[cit22] Wiinikka H., Gebart R., Boman C., Boström D., Nordin A., Öhman M. (2006). High-temperature aerosol formation in wood pellets flames: spatially resolved measurements. Combust. Flame.

[cit23] Garcia-Maraver A., Zamorano M., Fernandes U., Rabaçal M., Costa M. (2014). Relationship between fuel quality and gaseous and particulate matter emissions in a domestic pellet-fired boiler. Fuel.

[cit24] Lehtikangas P. (2001). Quality properties of pelletised sawdust, logging residues and bark. Biomass Bioenergy.

[cit25] Venturini E., Vassura I., Agostini F., Pizzi A., Toscano G., Passarini F. (2018). Effect of fuel quality classes on the emissions of a residential wood pellet stove. Fuel.

[cit26] Verma V. K., Bram S., Gauthier G., De Ruyck J. (2011). Performance of a domestic pellet boiler as a function of operational loads: part-2. Biomass Bioenergy.

[cit27] Venturini E., Vassura I., Zanetti C., Pizzi A., Toscano G., Passarini F. (2015). Evaluation of non-steady state condition contribution to the total emissions of residential wood pellet stove. Energy.

[cit28] Fachinger F., Drewnick F., Gieré R., Borrmann S. (2017). How the user can influence particulate emissions from residential wood and pellet stoves: emission factors for different fuels and burning conditions. Atmos. Environ..

[cit29] Reyes F., Vasquez Y., Gramsch E., Oyola P., Rappenglück B., Rubio M. A. (2019). Photooxidation of emissions from firewood and pellet combustion using a photochemical chamber. Atmosphere.

[cit30] Li C., Ma Z., Chen J., Wang X., Ye X., Wang L., Yang X., Kan H., Donaldson D. J., Mellouki A. (2015). Evolution of biomass burning smoke particles in the dark. Atmos. Environ..

[cit31] Tiitta P., Leskinen A., Hao L., Yli-Pirilä P., Kortelainen M., Grigonyte J., Tissari J., Lamberg H., Hartikainen A., Kuuspalo K., Kortelainen A.-M., Virtanen A., Lehtinen K. E. J., Komppula M., Pieber S., Prévôt A. S. H., Onasch T. B., Worsnop D. R., Czech H., Zimmermann R., Jokiniemi J., Sippula O. (2016). Transformation of logwood combustion emissions in a smog chamber: formation of secondary organic aerosol and changes in the primary organic aerosol upon daytime and nighttime aging. Atmos. Chem. Phys..

[cit32] Hartikainen A., Yli-Pirilä P., Tiitta P., Leskinen A., Kortelainen M., Orasche J., Schnelle-Kreis J., Lehtinen K. E. J., Zimmermann R., Jokiniemi J., Sippula O. (2018). Volatile organic compounds from logwood combustion: emissions and transformation under dark and photochemical aging conditions in a smog chamber. Environ. Sci. Technol..

[cit33] Kodros J. K., Papanastasiou D. K., Paglione M., Masiol M., Squizzato S., Florou K., Skyllakou K., Kaltsonoudis C., Nenes A., Pandis S. N. (2020). Rapid dark aging of biomass burning as an overlooked source of oxidized organic aerosol. Proc. Natl. Acad. Sci. U. S. A..

[cit34] Paglione M., Gilardoni S., Rinaldi M., Decesari S., Zanca N., Sandrini S., Giulianelli L., Bacco D., Ferrari S., Poluzzi V., Scotto F., Trentini A., Poulain L., Herrmann H., Wiedensohler A., Canonaco F., Prévôt A. S. H., Massoli P., Carbone C., Facchini M. C., Fuzzi S. (2020). The impact of biomass burning and aqueous-phase processing on air quality: a multi-year source apportionment study in the Po Valley, Italy. Atmos. Chem. Phys..

[cit35] Gilardoni S., Massoli P., Paglione M., Giulianelli L., Carbone C., Rinaldi M., Decesari S., Sandrini S., Costabile F., Gobbi G. P., Pietrogrande M. C., Visentin M., Scotto F., Fuzzi S., Facchini M. C. (2016). Direct observation of aqueous secondary organic aerosol from biomass-burning emissions. Proc. Natl. Acad. Sci. U. S. A..

[cit36] Duan J., Huang R.-J., Gu Y., Lin C., Zhong H., Xu W., Liu Q., You Y., Ovadnevaite J., Ceburnis D., Hoffmann T., O'Dowd C. (2022). Measurement report: large contribution of biomass burning and aqueous-phase processes to the wintertime secondary organic aerosol formation in Xi'an, Northwest China. Atmos. Chem. Phys..

[cit37] Jorga S. D., Florou K., Kaltsonoudis C., Kodros J. K., Vasilakopoulou C., Cirtog M., Fouqueau A., Picquet-Varrault B., Nenes A., Pandis S. N. (2021). Nighttime chemistry of biomass burning emissions in urban areas: a dual mobile chamber study. Atmos. Chem. Phys..

[cit38] Florou K., Papanastasiou D. K., Pikridas M., Kaltsonoudis C., Louvaris E., Gkatzelis G. I., Patoulias D., Mihalopoulos N., Pandis S. N. (2017). The contribution of wood burning and other pollution sources to wintertime organic aerosol levels in two Greek cities. Atmos. Chem. Phys..

[cit39] Saarikoski S., Carbone S., Decesari S., Giulianelli L., Angelini F., Canagaratna M., Ng N. L., Trimborn a., Facchini M. C., Fuzzi S., Hillamo R., Worsnop D. (2012). Chemical characterization of springtime submicrometer aerosol in Po Valley, Italy. Atmos. Chem. Phys..

[cit40] Crippa M., DeCarlo P. F., Slowik J. G., Mohr C., Heringa M. F., Chirico R., Poulain L., Freutel F., Sciare J., Cozic J., Di Marco C. F., Elsasser M., Nicolas J. B., Marchand N., Abidi E., Wiedensohler A., Drewnick F., Schneider J., Borrmann S., Nemitz E., Zimmermann R., Jaffrezo J.-L., Prévôt a. S. H., Baltensperger U. (2013). Wintertime aerosol chemical composition and source apportionment of the organic fraction in the metropolitan area of Paris. Atmos. Chem. Phys..

[cit41] Barmet P., Dommen J., DeCarlo P. F., Tritscher T., Praplan a. P., Platt S. M., Prévôt a. S. H., Donahue N. M., Baltensperger U. (2012). OH clock determination by proton transfer reaction mass spectrometry at an environmental chamber. Atmos. Meas. Tech..

[cit42] Jayne J. T., Leard D. C., Zhang X., Davidovits P., Smith K. A., Kolb C. E., Worsnop D. R. (2000). Development of an aerosol mass spectrometer for size and composition analysis of submicron particles. Aerosol Sci. Technol..

[cit43] Drewnick F., Hings S. S., DeCarlo P., Jayne J. T., Gonin M., Fuhrer K., Weimer S., Jimenez J. L., Demerjian K. L., Borrmann S., Worsnop D. R. (2005). A new time-of-flight aerosol mass spectrometer (TOF-AMS)—instrument description and first field deployment. Aerosol Sci. Technol..

[cit44] Canagaratna M. R., Jimenez J. L., Kroll J. H., Chen Q., Kessler S. H., Massoli P., Hildebrandt Ruiz L., Fortner E., Williams L. R., Wilson K. R., Surratt J. D., Donahue N. M., Jayne J. T., Worsnop D. R. (2015). Elemental ratio measurements of organic compounds using aerosol mass spectrometry: characterization, improved calibration, and implications. Atmos. Chem. Phys..

[cit45] Petzold A., Schönlinner M. (2004). Multi-angle absorption photometry—a new method for the measurement of aerosol light absorption and atmospheric black carbon. J. Aerosol Sci..

[cit46] An W. J., Pathak R. K., Lee B.-H., Pandis S. N. (2007). Aerosol volatility measurement using an improved thermodenuder: application to secondary organic aerosol. J. Aerosol Sci..

[cit47] Louvaris E. E., Florou K., Karnezi E., Papanastasiou D. K., Gkatzelis G. I., Pandis S. N. (2017). Volatility of source apportioned wintertime organic aerosol in the city of Athens. Atmos. Environ..

[cit48] Karnezi E., Riipinen I., Pandis S. N. (2014). Measuring the atmospheric organic aerosol volatility distribution: a theoretical analysis. Atmos. Meas. Tech..

[cit49] Riipinen I., Pierce J. R., Donahue N. M., Pandis S. N. (2010). Equilibration time scales of organic aerosol inside thermodenuders: evaporation kinetics versus thermodynamics. Atmos. Environ..

[cit50] Kaltsonoudis C., Kostenidou E., Florou K., Psichoudaki M., Pandis S. N. S. N. (2016). Temporal variability and sources of VOCs in urban areas of the eastern Mediterranean. Atmos. Chem. Phys..

[cit51] Yazdani A., Takahama S., Kodros J. K., Paglione M., Masiol M., Squizzato S., Florou K., Kaltsonoudis C., Jorga S. D., Pandis S. N., Nenes A. (2023). Chemical evolution of primary and secondary biomass burning aerosols during daytime and nighttime. Atmos. Chem. Phys..

[cit52] Kostenidou E., Pathak R. K., Pandis S. N. (2007). An algorithm for the calculation of secondary organic aerosol density combining AMS and SMPS data. Aerosol Sci. Technol..

[cit53] Wang N., Jorga S. D., Pierce J. R., Donahue N. M., Pandis S. N. (2018). Particle wall-loss correction methods in smog chamber experiments. Atmos. Meas. Tech..

[cit54] Pierce J. R., Engelhart G. J., Hildebrandt L., Weitkamp E. A., Pathak R. K., Donahue N. M., Robinson A. L., Adams P. J., Pandis S. N. (2008). Constraining particle evolution from wall losses, coagulation, and condensation-evaporation in smog-chamber experiments: optimal estimation based on size distribution measurements. Aerosol Sci. Technol..

[cit55] Donahue N. M., Robinson A. L., Stanier C. O., Pandis S. N. (2006). Coupled partitioning, dilution, and chemical aging of semivolatile organics. Environ. Sci. Technol..

[cit56] Kostenidou E., Lee B.-H., Engelhart G. J., Pierce J. R., Pandis S. N. (2009). Mass spectra deconvolution of low, medium, and high volatility biogenic secondary organic aerosol. Environ. Sci. Technol..

[cit57] Farmer D. K., Matsunaga A., Docherty K. S., Surratt J. D., Seinfeld J. H., Ziemann P. J., Jimenez J. L. (2010). Response of an aerosol mass spectrometer to organonitrates and organosulfates and implications for atmospheric chemistry. Proc. Natl. Acad. Sci. U. S. A..

[cit58] Fry J. L., Kiendler-Scharr A., Rollins A. W., Wooldridge P. J., Brown S. S., Fuchs H., Dubé W., Mensah A., dal Maso M., Tillmann R., Dorn H.-P., Brauers T., Cohen R. C. (2009). Organic nitrate and secondary organic aerosol yield from NO_3_ oxidation of β-pinene evaluated using a gas-phase kinetics/aerosol partitioning model. Atmos. Chem. Phys..

[cit59] Kiendler-Scharr A., Mensah A. A., Friese E., Topping D., Nemitz E., Prevot A. S. H., Äijälä M., Allan J., Canonaco F., Canagaratna M., Carbone S., Crippa M., Dall Osto M., Day D. A., De Carlo P., Di Marco C. F., Elbern H., Eriksson A., Freney E., Hao L., Herrmann H., Hildebrandt L., Hillamo R., Jimenez J. L., Laaksonen A., McFiggans G., Mohr C., O'Dowd C., Otjes R., Ovadnevaite J., Pandis S. N., Poulain L., Schlag P., Sellegri K., Swietlicki E., Tiitta P., Vermeulen A., Wahner A., Worsnop D., Wu H. C. (2016). Ubiquity of organic nitrates from nighttime chemistry in the European submicron aerosol. Geophys. Res. Lett..

[cit60] Liu T., Li Z., Chan M., Chan C. K. (2017). Formation of secondary organic aerosols from gas-phase emissions of heated cooking oils. Atmos. Chem. Phys..

[cit61] Nault B. A., Campuzano-Jost P., Day D. A., Schroder J. C., Anderson B., Beyersdorf A. J., Blake D. R., Brune W. H., Choi Y., Corr C. A., De Gouw J. A., Dibb J., Digangi J. P., Diskin G. S., Fried A., Gregory Huey L., Kim M. J., Knote C. J., Lamb K. D., Lee T., Park T., Pusede S. E., Scheuer E., Thornhill K. L., Woo J. H., Jimenez J. L. (2018). Secondary organic aerosol production from local emissions dominates the organic aerosol budget over Seoul, South Korea, during KORUS-AQ. Atmos. Chem. Phys..

[cit62] Kodros J. K., Kaltsonoudis C., Paglione M., Florou K., Jorga S., Vasilakopoulou C., Cirtog M., Cazaunau M., Picquet-Varrault B., Nenes A., Pandis S. N. (2022). Secondary aerosol formation during the dark oxidation of residential biomass burning emissions. Environ. Sci.: Atmos..

[cit63] Kakavas S., Pandis S. N., Nenes A. (2022). ISORROPIA-Lite: a comprehensive atmospheric aerosol thermodynamics module for Earth system models. Tellus B.

[cit64] Fountoukis C., Nenes A. (2007). ISORROPIA II: a computationally efficient thermodynamic equilibrium model for K^+^–Ca^2+^–Mg^2+^–NH_4_^+^–Na^+^–SO_4_^2−^–NO_3_^−^–Cl^−^–H_2_O aerosols. Atmos. Chem. Phys..

[cit65] Petters M. D., Kreidenweis S. M. (2007). A single parameter representation of hygroscopic growth and cloud condensation nucleus activity. Atmos. Chem. Phys..

[cit66] Psichoudaki M., Nenes A., Florou K., Kaltsonoudis C., Pandis S. N. (2018). Hygroscopic properties of atmospheric particles emitted during wintertime biomass burning episodes in Athens. Atmos. Environ..

[cit67] Guo H., Liu J., Froyd K. D., Roberts J. M., Veres P. R., Hayes P. L., Jimenez J. L., Nenes A., Weber R. J. (2017). Fine particle pH and gas–particle phase partitioning of inorganic species in Pasadena, California, during the 2010 CalNex campaign. Atmos. Chem. Phys..

[cit68] Ryu S. Y., Kim J. E., Zhuanshi H., Kim Y. J., Kang G. U. (2004). Chemical composition of post-harvest biomass burning aerosols in Gwangju, Korea. J. Air Waste Manage. Assoc..

[cit69] Pye H. O. T., Nenes A., Alexander B., Ault A. P., Barth M. C., Clegg S. L., Collett Jr J. L., Fahey K. M., Hennigan C. J., Herrmann H., Kanakidou M., Kelly J. T., Ku I.-T., McNeill V. F., Riemer N., Schaefer T., Shi G., Tilgner A., Walker J. T., Wang T., Weber R., Xing J., Zaveri R. A., Zuend A. (2020). The acidity of atmospheric particles and clouds. Atmos. Chem. Phys..

[cit70] Kuwata M., Zorn S. R., Martin S. T. (2012). Using elemental ratios to predict the density of organic material composed of carbon, hydrogen, and oxygen. Environ. Sci. Technol..

[cit71] McClure C. D., Lim C. Y., Hagan D. H., Kroll J. H., Cappa C. D. (2020). Biomass-burning-derived particles from a wide variety of fuels – part 1: properties of primary particles. Atmos. Chem. Phys..

[cit72] Stockwell C. E., Veres P. R., Williams J., Yokelson R. J. (2015). Characterization of biomass burning emissions from cooking fires, peat, crop residue, and other fuels with high-resolution proton-transfer-reaction time-of-flight mass spectrometry. Atmos. Chem. Phys..

[cit73] Newland M. J., Ren Y., McGillen M. R., Michelat L., Daële V., Mellouki A. (2022). NO_3_ chemistry of wildfire emissions: a kinetic study of the gas-phase reactions of furans with the NO_3_ radical. Atmos. Chem. Phys..

[cit74] Coggon M. M., Veres P. R., Yuan B., Koss A., Warneke C., Gilman J. B., Lerner B. M., Peischl J., Aikin K. C., Stockwell C. E., Hatch L. E., Ryerson T. B., Roberts J. M., Yokelson R. J., de Gouw J. A. (2016). Emissions of nitrogen-containing organic compounds from the burning of herbaceous and arboraceous biomass: fuel composition dependence and the variability of commonly used nitrile tracers. Geophys. Res. Lett..

[cit75] Kari E., Hao L., Yli-Pirilä P., Leskinen A., Kortelainen M., Grigonyte J., Worsnop D. R., Jokiniemi J., Sippula O., Faiola C. L., Virtanen A. (2017). Effect of pellet boiler exhaust on secondary organic aerosol formation from α-pinene. Environ. Sci. Technol..

[cit76] Yokelson R. J., Griffith D. W. T., Ward D. E. (1996). Open-path Fourier transform infrared studies of large-scale laboratory biomass fires. J. Geophys. Res.: Atmos..

[cit77] Ward D. E., Hardy C. C. (1991). Smoke emissions from wildland fires. Environ. Int..

[cit78] Johansson L. S., Tullin C., Leckner B., Sjövall P. (2003). Particle emissions from biomass combustion in small combustors. Biomass Bioenergy.

[cit79] Zhao X., Wang L. (2017). Atmospheric Oxidation Mechanism of Furfural Initiated by Hydroxyl Radicals. J. Phys. Chem. A.

[cit80] Arora P. K. (1986). Chemiluminescent reactions of ozone with 2,5-dimethylfuran, furfural and pyrrole. J. Photochem..

[cit81] Ng N. L., Canagaratna M. R., Jimenez J. L., Chhabra P. S., Seinfeld J. H., Worsnop D. R. (2011). Changes in organic aerosol composition with aging inferred from aerosol mass spectra. Atmos. Chem. Phys..

[cit82] Hodzic A., Jimenez J. L., Madronich S., Aiken A. C., Bessagnet B., Curci G., Fast J., Lamarque J.-F., Onasch T. B., Roux G., Schauer J. J., Stone E. A., Ulbrich I. M. (2009). Modeling organic aerosols during MILAGRO: importance of biogenic secondary organic aerosols. Atmos. Chem. Phys..

[cit83] Rollins A. W., Kiendler-Scharr A., Fry J. L., Brauers T., Brown S. S., Dorn H.-P., Dubé W. P., Fuchs H., Mensah A., Mentel T. F., Rohrer F., Tillmann R., Wegener R., Wooldridge P. J., Cohen R. C. (2009). Isoprene oxidation by nitrate radical: alkyl nitrate and secondary organic aerosol yields. Atmos. Chem. Phys..

[cit84] Palm B. B., Peng Q., Fredrickson C. D., Lee B. H., Garofalo L. A., Pothier M. A., Kreidenweis S. M., Farmer D. K., Pokhrel R. P., Shen Y., Murphy S. M., Permar W., Hu L., Campos T. L., Hall S. R., Ullmann K., Zhang X., Flocke F., Fischer E. V., Thornton J. A. (2020). Quantification of organic aerosol and brown carbon evolution in fresh wildfire plumes. Proc. Natl. Acad. Sci. U. S. A..

[cit85] Simoneit B. R. T., Schauer J. J., Nolte C. G., Oros D. R., Elias V. O., Fraser M. P., Rogge W. F., Cass G. R. (1999). Atmos. Environ..

[cit86] Cubison M. J., Ortega a. M., Hayes P. L., Farmer D. K., Day D., Lechner M. J., Brune W. H., Apel E., Diskin G. S., Fisher J. a., Fuelberg H. E., Hecobian A., Knapp D. J., Mikoviny T., Riemer D., Sachse G. W., Sessions W., Weber R. J., Weinheimer a. J., Wisthaler A., Jimenez J. L. (2011). Effects of aging on organic aerosol from open biomass burning smoke in aircraft and laboratory studies. Atmos. Chem. Phys..

[cit87] Weimer S., Alfarra M. R., Schreiber D., Mohr M., Prévôt A. S. H. H., Baltensperger U. (2008). Organic aerosol mass spectral signatures from wood-burning emissions: influence of burning conditions and wood type. J. Geophys. Res..

[cit88] Li Y. J., Yeung J. W. T., Leung T. P. I., Lau A. P. S., Chan C. K. (2012). Characterization of Organic Particles from Incense Burning Using an Aerodyne High-Resolution Time-of-Flight Aerosol Mass Spectrometer. Aerosol Sci. Technol..

[cit89] Ulbrich I. M., Canagaratna M. R., Zhang Q., Worsnop D. R., Jimenez J. L. (2009). Interpretation of organic components from Positive Matrix Factorization of aerosol mass spectrometric data. Atmos. Chem. Phys..

[cit90] Ge X., Setyan A., Sun Y., Zhang Q. (2012). Primary and secondary organic aerosols in Fresno, California during wintertime: results from high resolution aerosol mass spectrometry. J. Geophys. Res..

[cit91] Saarikoski S., Reyes F., Vázquez Y., Tagle M., Timonen H., Aurela M., Carbone S., Worsnop D. R., Hillamo R., Oyola P. (2019). Characterization of submicron aerosol chemical composition and sources in the coastal area of Central Chile. Atmos. Environ..

[cit92] Casotto R., Cvitešić Kušan A., Bhattu D., Cui T., Manousakas M. I., Frka S., Kroflič A., Grgić I., Ciglenečki I., Baltensperger U., Slowik J. G., Daellenbach K. R., Prévôt A. S. H. (2022). Chemical composition and sources of organic aerosol on the Adriatic coast in Croatia. Atmos. Environ.: X.

