# Supplementary material for: Characterization and dark oxidation of the emissions of a pellet stove
Source: Environ Sci Atmos. 2023 Jul 28;3(9):1319–34. doi: 10.1039/d3ea00070b (PMC10500314; doi:10.1039/d3ea00070b)
Supplement: EA-003-D3EA00070B-s001 [file EA-003-D3EA00070B-s001.pdf]

Electronic Supplementary Material (ESI) for Environmental Science: Atmospheres.
This journal is © The Royal Society of Chemistry 2023

Characterization and dark oxidation of the emissions of a pellet stove

Kalliopi Florou[1], John K. Kodros[1], Marco Paglione[1,2], Spiro Jorga[3], Stefania Squizzato[1], Mauro Masiol[1,4], Petro Uruci[1,5], Athanasios Nenes[1,6*], Spyros N. Pandis[1,5*]

[1]Institute of Chemical Engineering Sciences, ICE−HT, Patras, 26504, Greece

[2]Institute of Atmospheric Sciences and Climate, Italian National Research Council, Bologna 40129, Italy

[3]Department of Chemical Engineering, Carnegie Mellon University, Pittsburgh, 15213, USA

[4]Department of Environmental Sciences, Informatics and Statistics, Università Ca' Foscari Venezia, Venice, Italy

[5]Department of Chemical Engineering, University of Patras, Patras 26504, Greece

[6]School of Architecture, Civil and Environmental Engineering, Swiss Federal Institute of Technology Lausanne, Lausanne 1015, Switzerland

Correspondence to: Spyros N. Pandis (spyros@chemeng.upatras.gr), Athanasios Nenes (athanasios.nenes@epfl.ch)

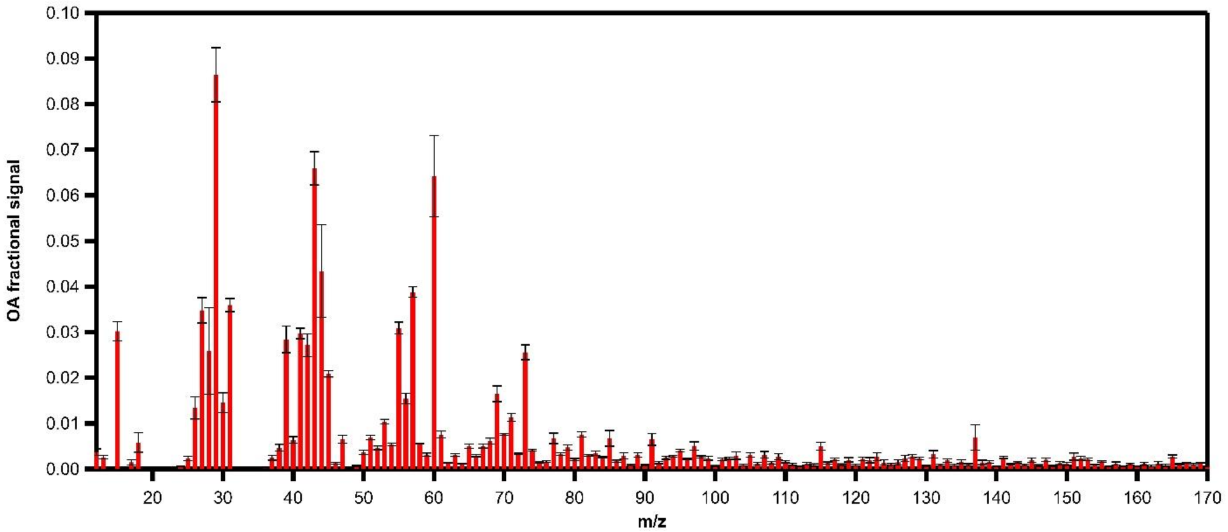

Figure S1. Average OA mass spectra of fresh pellet emissions (red bars) and standard deviation (black error bars) from all experiments.

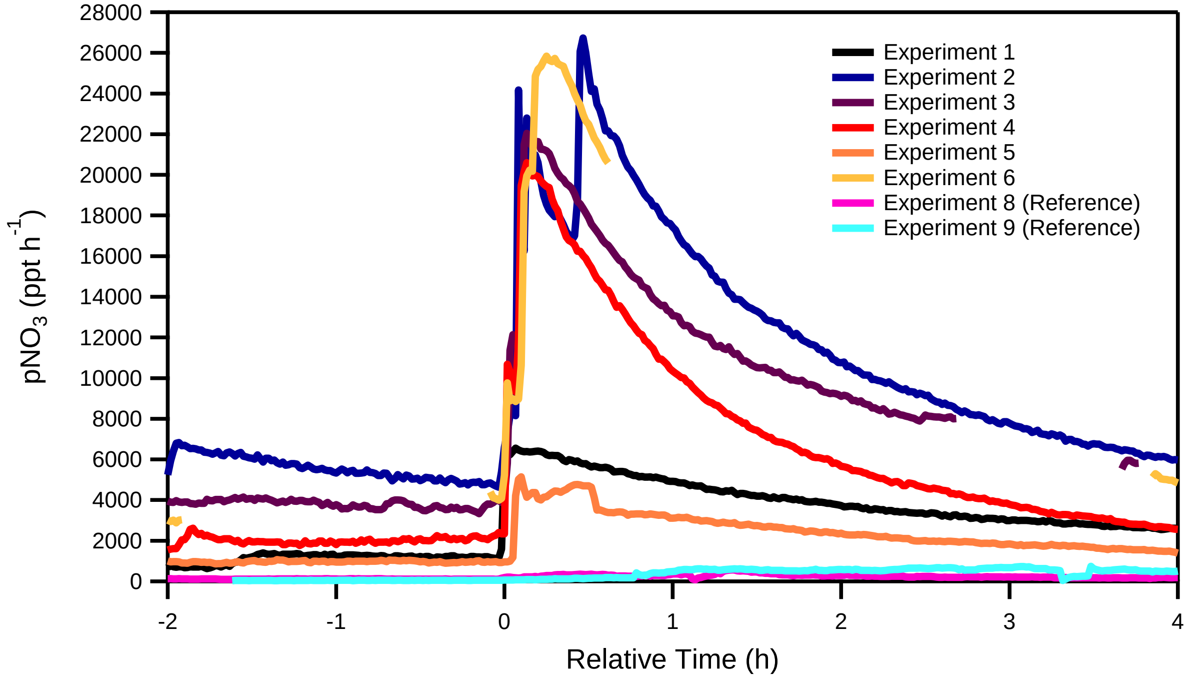

Figure S2. Production of nitrate radicals in ppt per h, assuming only NO_2+O_3 reaction.

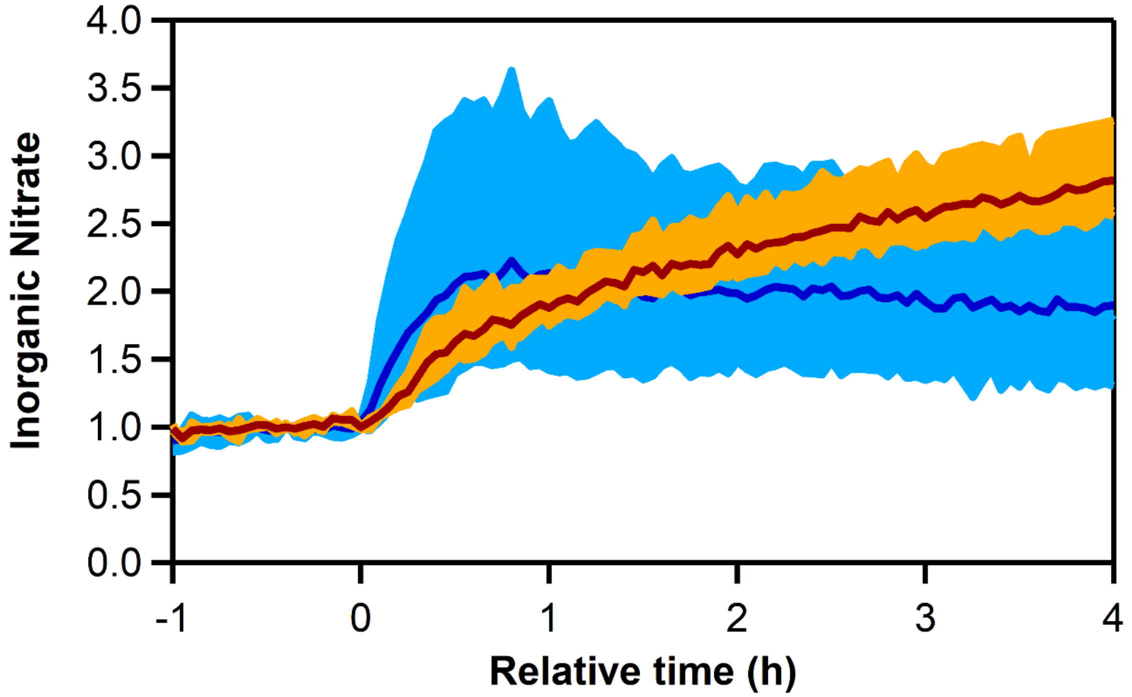

Figure S3. Fractional enhancement of inorganic nitrate for the dark aging experiments under dry (brown line; experiments 1 to 3) and high RH (blue line; experiments 4 to 6). The shaded light blue (dark high RH experiments) and orange (dark dry experiments) regions correspond to the variability across all experiments due to differences in injected NO_2 and O_3 concentration, while the solid blue and brown lines are the mean across the RH and dry experiments, respectively.

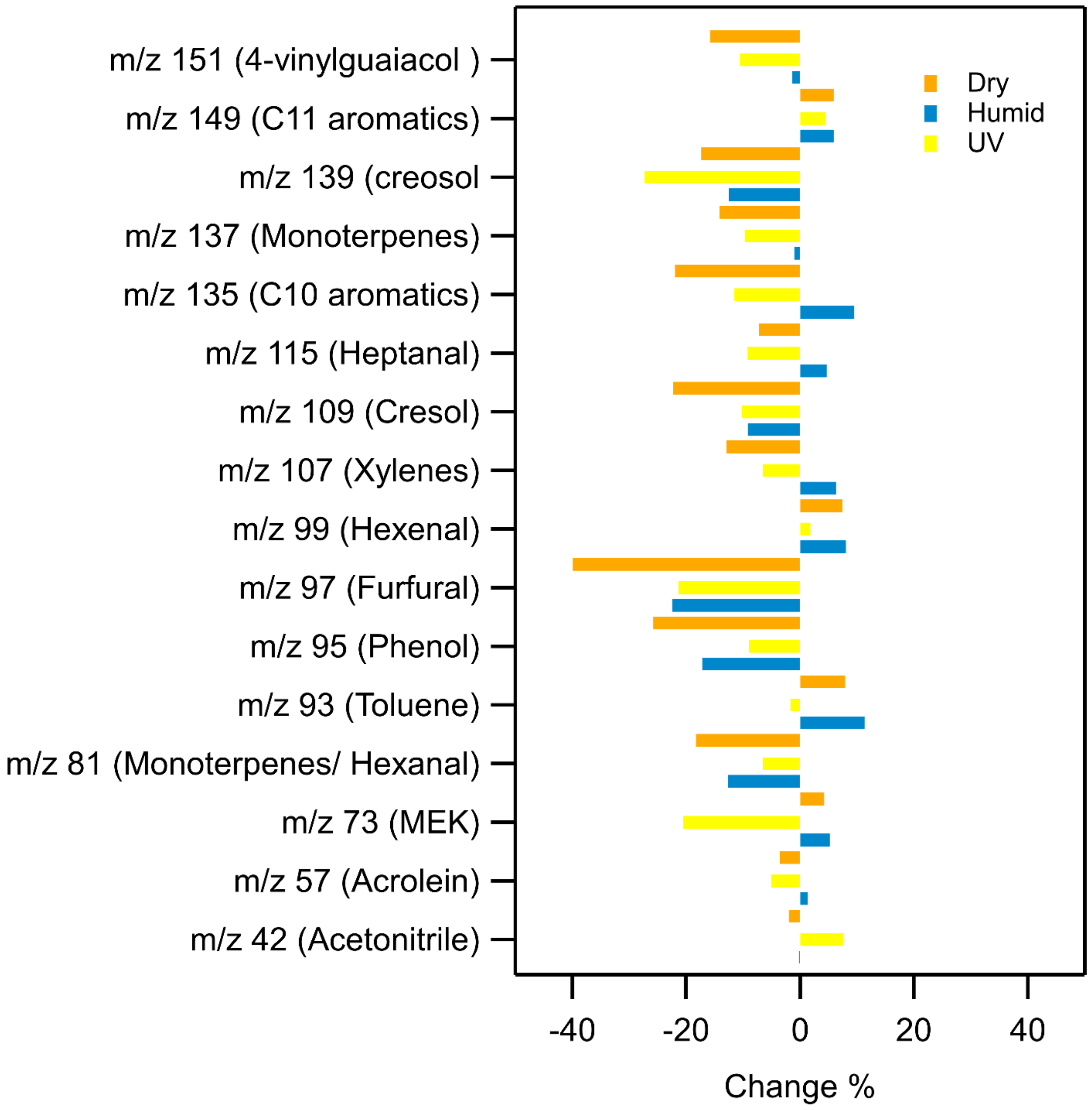

Figure S4. Change in concentration (in %) of certain VOCs for UV, humid and low RH experiments after aging.

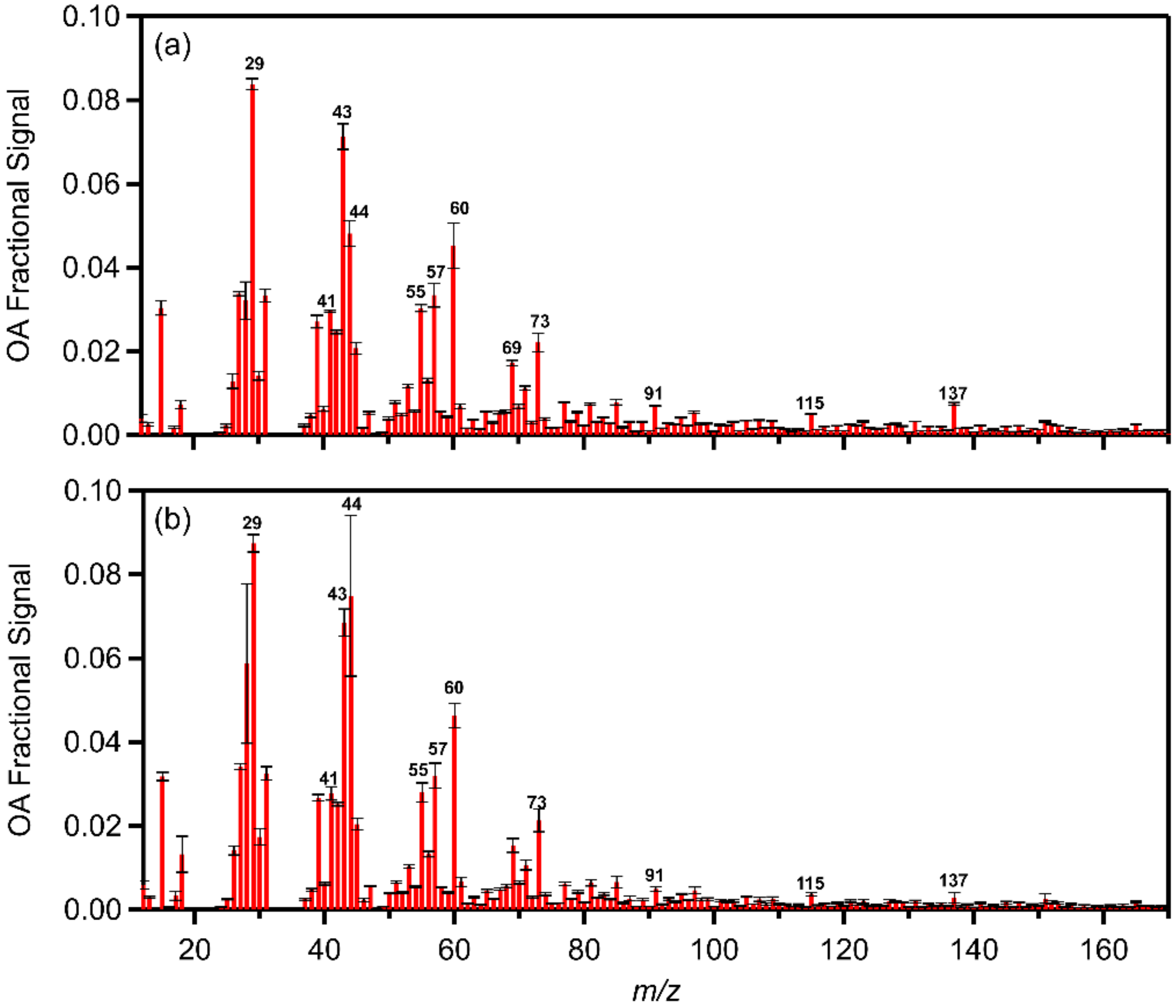

Figure S5. Average OA mass spectra of aged pellet emissions (red bars) and standard deviation (black error bars) for a) low and b) high RH experiments.

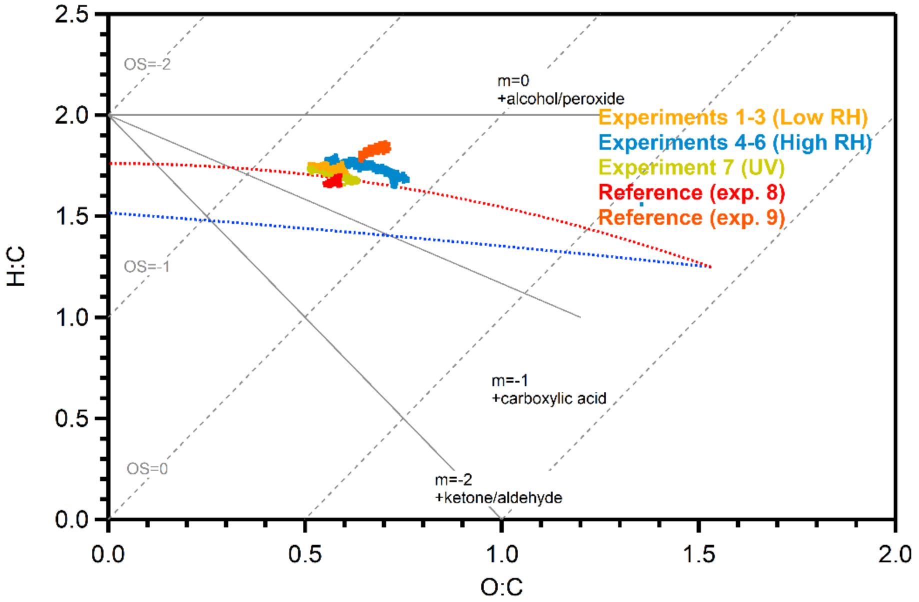

Figure S6. The Van Krevelen (VK) triangle diagram presents the relation of the H:C and O:C ratio for the pellet-burning experiments shown here. The OA components from this dataset mostly fall outside the VK-triangle region.[1]

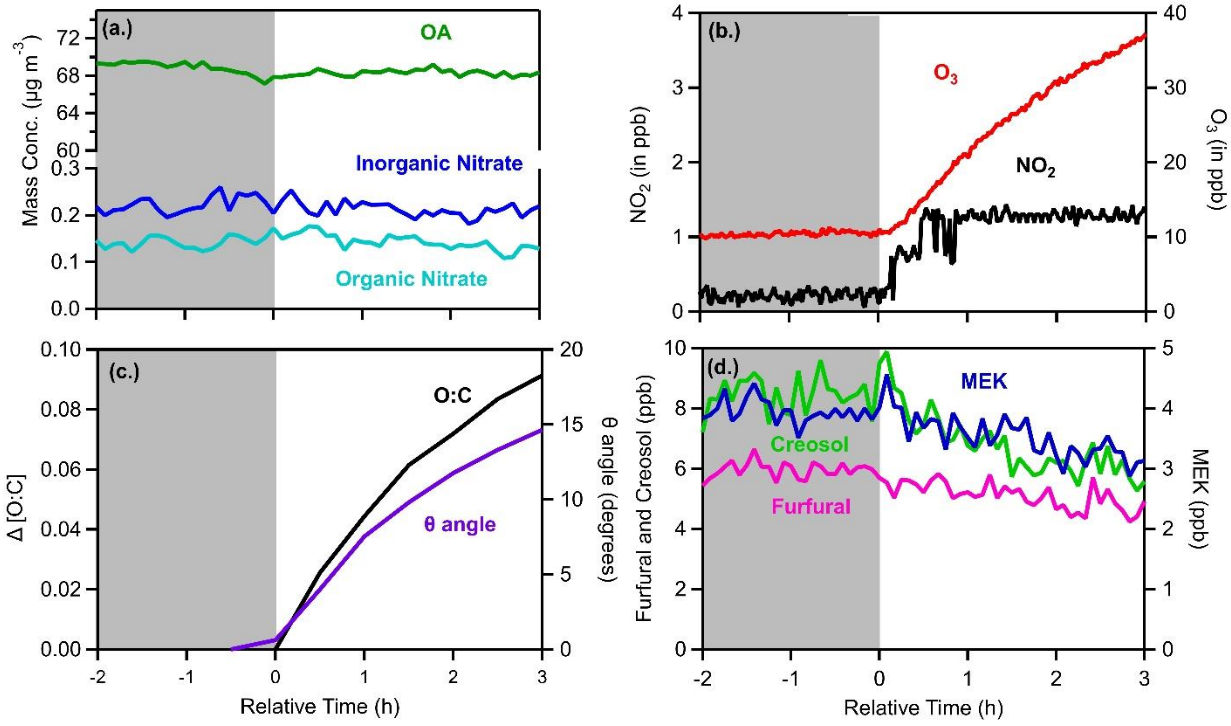

Figure S7. Measurements for the photooxidation (Experiment 7 in Table 1): **a)** wall-loss corrected OA, PM inorganic nitrate, and PM organic nitrate, **b)** gas-phase species NO_2, and O_3, **c)** the change in the O:C ratio and theta angle **d)** representative VOCs showing the largest decrease (MEK, *m/z* 73; furfural, 97 and creosol, *m/z* 139).

Aerosol acidity

Four different cases have been simulated and are shown here: a) Base case: measured OA and a potassium concentration estimated as 15% of the PM nitrate [2]; b) both OA and potassium concentrations were assumed to be zero; c) measured OA and a zero assumed potassium concentration; d) a zero assumed OA and scaled potassium concentrations.

For the high RH experiments, the different sensitivity tests showed that, when including neither OA water uptake nor potassium in the model (case b), the pH of the pellet aerosol stayed constant throughout the experiment for all cases with an average value of 2.8 ± 0.3. This is consistent with the fact that the inorganic components of the BB aerosol (which control the acidity in this case) is not affected by the aging process. When OA water uptake alone is included (case c), the pH of the OA decreased after ozone injection, but only in experiment 4 (Figure S5a) from 3.8 to 3.1, while for experiments 5 and 6 pH was constant and equal to 3.1 for both. This indicates that the water uptake from organics in many of the experiments is not significant enough to have a large impact on nitrate/ammonium partitioning and ozone injection did not affect it. Similarly, when an estimate of potassium emissions is included (but without OA water uptake; case d), the pH decreases after t=0 h, from 3.6 to 3.2 only in the case of exp. 4, while for the other two high RH experiments, pH remains constant at 2.9 ± 0.1.

In the low RH cases, when including neither OA water uptake nor potassium in the model (case b), the pH of the pellet aerosol stays constant for the whole experiment, and the same applies for exp. 7 (UV ref.), in which pH equals 1 even after the lights are turned on. When OA water uptake alone is included (case c), the pH of the OA increased after ozone injection for all three dark dry cases – owing to the dilution effect that the organic water has on acidity. For experiments 2 and 3 (where ozone and NO_2 injections were higher than 100 ppb) pH increased for both from 2.7 to 3.7, while in experiment 1 (under lower oxidants conditions) increased from 2.5 to 3.1. In the case where only scaled potassium is included (case d), pH remained constant for the whole experiment – reflecting that the amount and partitioning of inorganics is largely unaffected by the aging process.

The simulated inorganic nitrate matches the observations for the whole experiment for the base case simulation (OA water uptake and potassium are included) (Exp.4; Figure 5b), which confirms that the pH estimates here are realistic. When the OA water uptake and potassium are neglected, nitrate is underestimated by a factor of 2, but follows the trend of observations – which points to the need of including organic water in partitioning and pH calculations. Finally, when only potassium is included, inorganic nitrate is underestimated only for the period before the ozone injection and matches the measurements right after the start of dark oxidation.

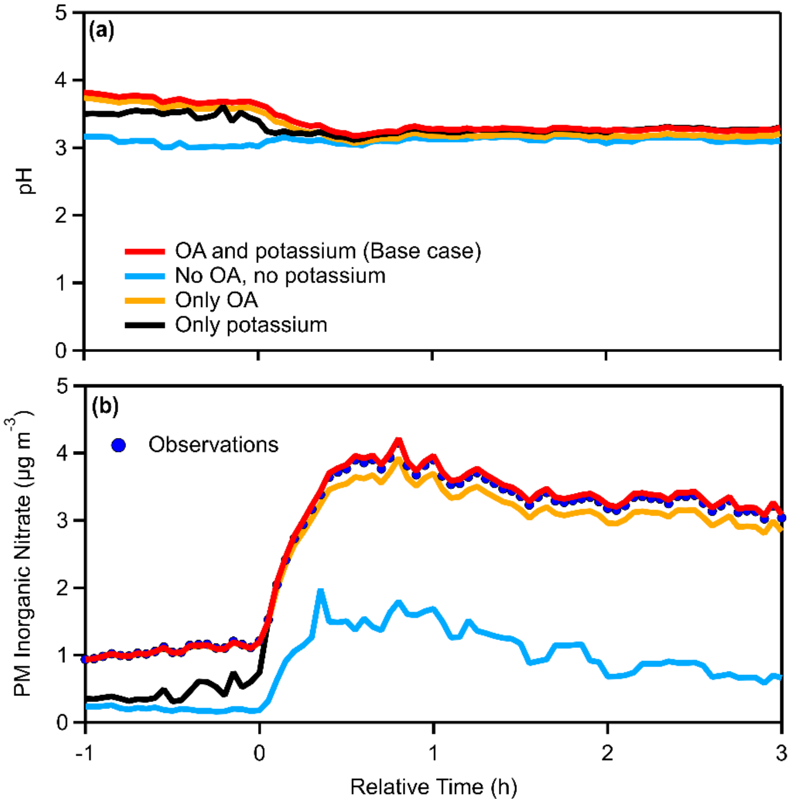

Figure S8. Estimated (a) aerosol pH for experiment 4 including sensitivity simulations for base case (red), neither OA water uptake nor K^+ (light blue), only OA water uptake (orange), and only potassium (black), and (c) PM inorganic nitrate for experiment 4 for the different simulations, with measurements from the HR-ToF-AMS presented as blue dots.

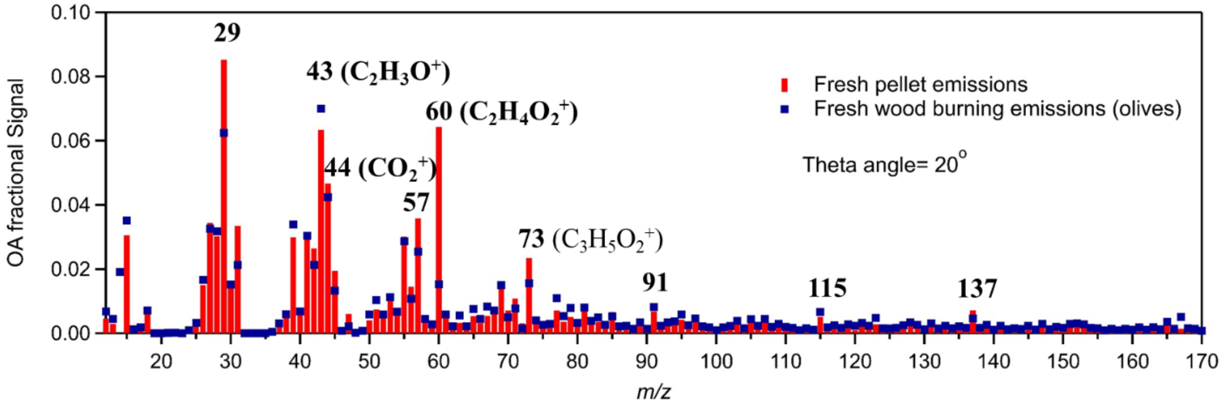

Figure S9. Comparison of fresh pellets (red bars; this work) and olive wood (blue squares; Kodros et al. [3]) spectra.

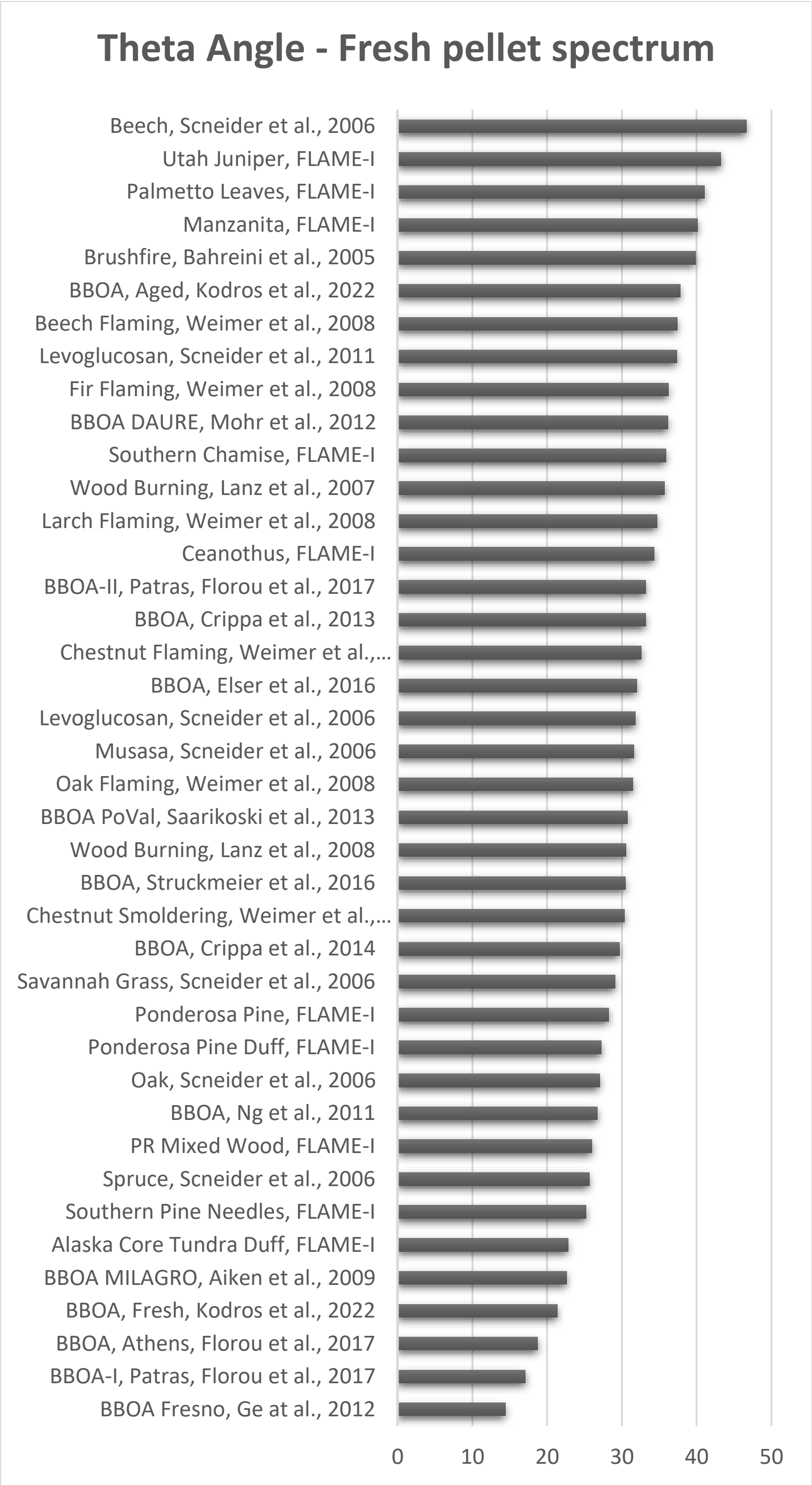

Figure S10. Theta angles between the fresh pellet mass spectrum and BBOA/ wood spectra from the literature.

(database: Ulbrich, I.M., Handschy, A., Lechner, M., and Jimenez, J.L. High-Resolution AMS Spectral Database. URL: http://cires.colorado.edu/jimenez-group/HRAMSsd/) [1,3–16]

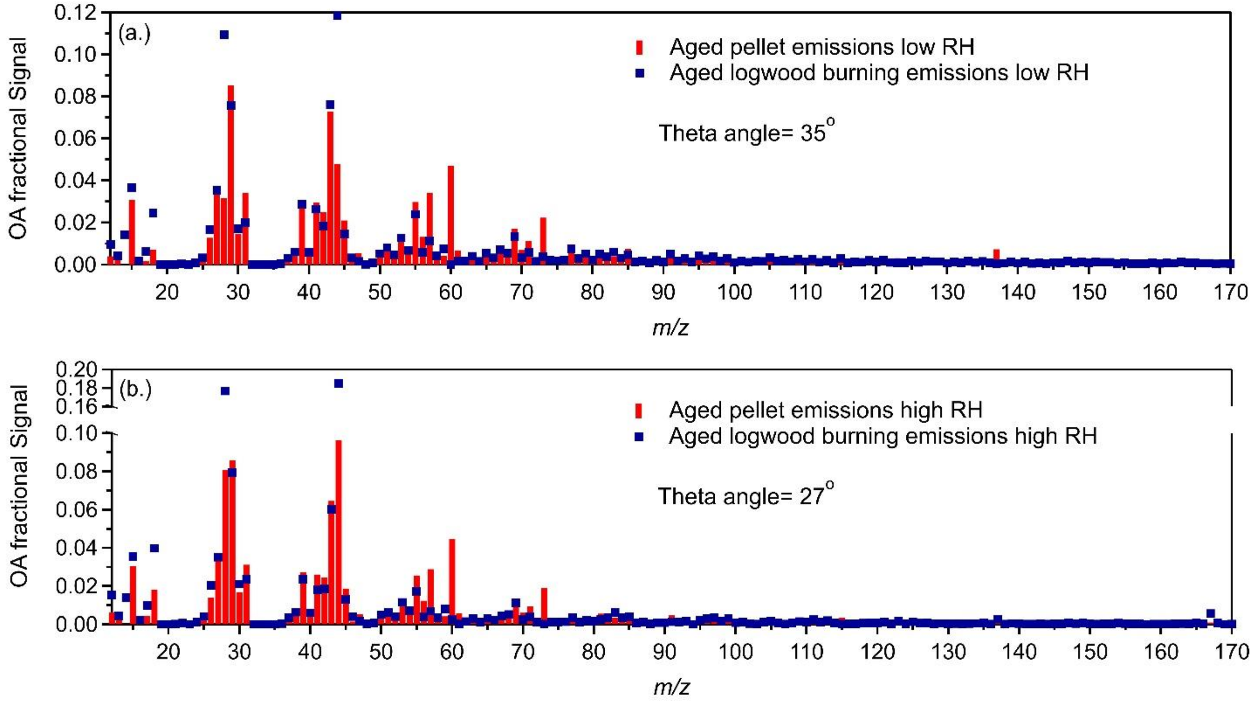

Figure S11. Comparison of aged pellets (red bars; this work) and olive wood (blue squares; Kodros et al. [3]) spectra for a) low RH and b) high RH conditions.

Table S1. Theta angle of HR spectra during the emissions period.

Emissions:	Exp.1	Exp.2	Exp.3	Exp.4	Exp.5	Exp.6
Exp.2	8					
Exp.3	5	4				
Exp.4	5	9	6			
Exp.5	6	4	3	6		
Exp.6	12	14	13	9	12	
Exp.7	11	18	15	11	16	12

Table S2. Assumed reaction rate constants used to calculate typical lifetime of the VOCs with the largest observed decrease. Reaction rate constants are taken from the listed publications and the Master Chemical Mechanism, MCM v3.3.1[17].

Oxidant	$k_{furfural}$	k_{phenol}	$k_{creosol}$	k_{cresol}	$k_{a\text{-}pinene}$
	(molecule^{-1} cm^{3} s^{-1})				
NO_3	9.07 x 10^{-14}	3.92 x 10^{-12}	2.4 x 10^{-13}	1.4 x 10^{-13}	2−4 x 10^{-12}
	(Newland et al., 2022) [18]	(Atkinson et al., 1992) [19]	(Yang et al., 2016) [20]	(Atkinson et al., 1992) [19]	(MCM v3.3.1)
	1.17 x 10^{-12}				
	(Colmenar et al., 2012) [21]				
OH	3.51 x 10^{-11}	2.63 x 10^{-11}	9.51 x 10^{-11}	4.3−5.9 x 10^{-11}	3.9 x 10^{-12}<br>3.0 x 10^$^{-11}$
	(Bierbach et al., 1995) [22]	(Atkinson et al., 1992) [19]	(Coeur-Tourneur et al., 2010) [23]	(Coeur-Tourneur et al., 2010) [23]	(MCM v3.3.1)

Table S3. Average concentrations of oxidants and estimated lifetimes for the VOCs in Experiment 1.

Oxidant	Concentration	$\tau_{furfural}$	τ_{phenol}	$\tau_{creosol}$	τ_{cresol}	$\tau_{a\text{-}pinene}$
	(molecule cm^{-3})	(h)				
NO_3	5 x 10^{8}	0.5 – 6.1	0.14	2.3	4	0.28
	8 x 10^{8}	0.3 − 3.8	0.1	1.4	2.5	0.1
OH	1.4 x 10^{6}	5.7	7.5	2.1	3.4 – 4.6	6.6 − 51

Table S4. Theta angle of HR spectra for the 4 h period (aged).

After 4 h:	Exp.1	Exp.2	Exp.3	Exp.4	Exp.5	Exp.6
Exp.2	8					
Exp.3	5	4				
Exp.4	12	17	15			
Exp.5	5	7	5	11		
Exp.6	19	24	22	8	18	
Exp.7	18	25	23	9	20	8

Table S5. Calculated pH for emissions and after 4 h of time zero.

	pH fresh	pH after 4 h
Exp.1	3.0	3.6
Exp.2	3.2	4.0
Exp.3	3.1	4.1
Exp.4	3.8	3.3
Exp.5	3.2	3.0
Exp.6	3.2	3.1
Exp.7	2.5	2.8
Average (exp.1-6)	3.2	3.5
Average (low RH only)	3.1	3.9
Average (high RH only)	3.4	3.1

Table S6. Comparisons (quantified by the theta angle in degrees) of dark- dry and humid pellet spectra from this study to AMS OA factors from literature. The same spectra are used for Fig. 9 in the main text.

**Reference**	**Location**	**Factor**	**Dark-Dry**	**Dark-Humid**
Saarikoski et al. (2012)[15]	Po Valley, Italy	BBOA	31	38
Florou et al. (2017)[4]	Athens, Greece	BBOA	16	24
Florou et al. (2017)[4]	Patras, Greece	BBOA-I	13	23
Florou et al. (2017)[4]	Patras, Greece	BBOA-II	31	29
Elser et al. (2016)[13]	Beijing, China	BBOA	29	40
Mohr et al. (2012)[14]	Barcelona, Spain	BBOA DAURE	32	38
Struckmeier et al. (2016)[6]	Rome, Italy	BBOA	27	25
Ge et al. (2012)[7]	Fresno, USA	BBOA	13	17
Florou et al. (2017)[4]	Patras, Greece	OOA	43	30
Florou et al. (2017)[4]	Athens, Greece	OOA	39	28
Saarikoski et al. (2012)[15]	Po Valley, Italy	OOAa *	49	36
Saarikoski et al. (2012)[15]	Po Valley, Italy	OOAb	41	28
Saarikoski et al. (2012)[15]	Po Valley, Italy	OOAc *	50	37
Docherty et al. (2011)[24]	Riverside, USA	SV-OOA SOAR	29	30
Sun et al. (2011)[25]	NY, USA	OOA	40	30
Crippa et al. (2013)[16]	Paris, France	OOA-BBOA2	28	20
Ge et al. (2012)[7]	Fresno, USA	OOA	30	22
Mohr et al. (2012)[14]	Barcelona, Spain	SV-OOA DAURE	31	26

*It is not shown in Figure 9.

References:

1 N. L. Ng, M. R. Canagaratna, J. L. Jimenez, P. S. Chhabra, J. H. Seinfeld and D. R. Worsnop, Changes in organic aerosol composition with aging inferred from aerosol mass spectra, *Atmos. Chem. Phys.*, 2011, **11**, 6465–6474.

2 S. Y. Ryu, J. E. Kim, H. Zhuanshi, Y. J. Kim and G. U. Kang, Chemical composition of post-harvest biomass burning aerosols in Gwangju, Korea, *J. Air Waste Manag. Assoc.*, 2004, **54**, 1124–1137.

3 J. K. Kodros, C. Kaltsonoudis, M. Paglione, K. Florou, S. Jorga, C. Vasilakopoulou, M. Cirtog, M. Cazaunau, B. Picquet-Varrault, A. Nenes and S. N. Pandis, Secondary aerosol formation during the dark oxidation of residential biomass burning emissions, *Environ. Sci. Atmos.*, 2022, **2**, 1221–1236.

4 K. Florou, D. K. Papanastasiou, M. Pikridas, C. Kaltsonoudis, E. Louvaris, G. I. Gkatzelis, D. Patoulias, N. Mihalopoulos and S. N. Pandis, The contribution of wood burning and other pollution sources to wintertime organic aerosol levels in two Greek cities, *Atmos. Chem. Phys.*, 2017, **17**, 3145–3163.

5 A. C. Aiken, D. Salcedo, M. J. Cubison, J. A. Huffman, P. F. DeCarlo, I. M. Ulbrich, K. S. Docherty, D. Sueper, J. R. Kimmel, D. R. Worsnop, A. Trimborn, M. Northway, E. A. Stone, J. J. Schauer, R. M. Volkamer, E. Fortner, B. de Foy, J. Wang, A. Laskin, V. Shutthanandan, J. Zheng, R. Zhang, J. Gaffney, N. A. Marley, G. Paredes-Miranda, W. P. Arnott, L. T. Molina, G. Sosa and J. L. Jimenez, Mexico City aerosol analysis during MILAGRO using high resolution aerosol mass spectrometry at the urban supersite (T0) – Part 1: Fine particle composition and organic source apportionment, *Atmos. Chem. Phys.*, 2009, **9**, 6633–6653.

6 C. Struckmeier, F. Drewnick, F. Fachinger, G. P. Gobbi and S. Borrmann, Atmospheric aerosols in Rome, Italy: sources, dynamics and spatial variations during two seasons, *Atmos. Chem. Phys.*, 2016, **16**, 15277–15299.

7 X. Ge, A. Setyan, Y. Sun and Q. Zhang, Primary and secondary organic aerosols in Fresno, California during wintertime: Results from high resolution aerosol mass spectrometry, *J. Geophys. Res. Atmos.*, 2012, **117**, n/a-n/a.

8 I. M. Ulbrich, M. R. Canagaratna, Q. Zhang, D. R. Worsnop and J. L. Jimenez, Interpretation of organic components from Positive Matrix Factorization of aerosol mass spectrometric data, *Atmos. Chem. Phys.*, 2009, **9**, 2891–2918.

9 S. Weimer, M. R. Alfarra, D. Schreiber, M. Mohr, A. S. H. H. Prévôt and U. Baltensperger, Organic aerosol mass spectral signatures from wood-burning emissions: Influence of burning conditions and wood type, *J. Geophys. Res.*, 2008, **113**, 1–10.

10 J. Schneider, S. Weimer, F. Drewnick, S. Borrmann, G. Helas, P. Gwaze, O. Schmid, M. O. Andreae and U. Kirchner, Mass spectrometric analysis and aerodynamic properties of various types of combustion-related aerosol particles, *Int. J. Mass Spectrom.*, 2006, **258**, 37–49.

11 J. Schneider, F. Freutel, S. R. Zorn, Q. Chen, D. K. Farmer, J. L. Jimenez, S. T. Martin, P. Artaxo, A. Wiedensohler and S. Borrmann, Mass-spectrometric identification of primary biological particle markers and application to pristine submicron aerosol measurements in Amazonia, *Atmos. Chem. Phys.*, 2011, **11**, 11415–11429.

12 V. A. Lanz, M. R. Alfarra, U. Baltensperger, B. Buchmann, C. Hueglin and A. S. H. Prévôt, Source apportionment of submicron organic aerosols at an urban site by factor analytical modelling of aerosol mass spectra, *Atmos. Chem. Phys.*, 2007, **7**, 1503–1522.

13 M. Elser, R.-J. Huang, R. Wolf, J. G. Slowik, Q. Wang, F. Canonaco, G. Li, C. Bozzetti, K. R. Daellenbach, Y. Huang, R. Zhang, Z. Li, J. Cao, U. Baltensperger, I. El-Haddad and A. S. H. Prévôt, New insights into PM_{2.5} chemical composition and sources in two major cities in China during extreme haze events using aerosol mass spectrometry, *Atmos. Chem. Phys.*, 2016, **16**, 3207–3225.

14 C. Mohr, P. F. DeCarlo, M. F. Heringa, R. Chirico, J. G. Slowik, R. Richter, C. Reche, a. Alastuey, X. Querol, R. Seco, J. Peñuelas, J. L. Jiménez, M. Crippa, R. Zimmermann, U. Baltensperger and a. S. H. Prévôt, Identification and quantification of organic aerosol from cooking and other sources in Barcelona using aerosol mass spectrometer data, *Atmos. Chem. Phys.*, 2012, **12**, 1649–1665.

15 S. Saarikoski, S. Carbone, S. Decesari, L. Giulianelli, F. Angelini, M. Canagaratna, N. L. Ng, a. Trimborn, M. C. Facchini, S. Fuzzi, R. Hillamo and D. Worsnop, Chemical characterization of springtime submicrometer aerosol in Po Valley, Italy, *Atmos. Chem. Phys.*, 2012, **12**, 8401–8421.

16 M. Crippa, I. El Haddad, J. G. Slowik, P. F. DeCarlo, C. Mohr, M. F. Heringa, R. Chirico, N. Marchand, J. Sciare, U. Baltensperger and A. S. H. Prévôt, Identification of marine and continental aerosol sources in Paris using high resolution aerosol mass spectrometry, *J. Geophys. Res. Atmos.*, 2013, **118**, 1950–1963.

17 M. E. Jenkin, J. C. Young and A. R. Rickard, The MCM v3.3.1 degradation scheme for isoprene, *Atmos. Chem. Phys.*, 2015, **15**, 11433–11459.

18 M. J. Newland, Y. Ren, M. R. McGillen, L. Michelat, V. Daële and A. Mellouki, NO3 chemistry of wildfire emissions: a kinetic study of the gas-phase reactions of furans with the NO3 radical, *Atmos. Chem. Phys.*, 2022, **22**, 1761–1772.

19 R. Atkinson, S. M. Aschmann and J. Arey, Reactions of hydroxyl and nitrogen trioxide

radicals with phenol, cresols, and 2-nitrophenol at 296 .+-. 2 K, *Environ. Sci. Technol.*, 1992, **26**, 1397–1403.

20 B. Yang, H. Zhang, Y. Wang, P. Zhang, J. Shu, W. Sun and P. Ma, Experimental and theoretical studies on gas-phase reactions of NO3 radicals with three methoxyphenols: Guaiacol, creosol, and syringol, *Atmos. Environ.*, 2016, **125**, 243–251.

21 I. Colmenar, B. Cabañas, E. Martínez, M. S. Salgado and P. Martín, Atmospheric fate of a series of furanaldehydes by their NO3 reactions, *Atmos. Environ.*, 2012, **54**, 177–184.

22 A. Bierbach, I. Barnes and K. H. Becker, Product and kinetic study of the oh-initiated gas-phase oxidation of Furan, 2-methylfuran and furanaldehydes at ≈ 300 K, *Atmos. Environ.*, 1995, **29**, 2651–2660.

23 C. Coeur-Tourneur, A. Cassez and J. C. Wenger, Rate Coefficients for the Gas-Phase Reaction of Hydroxyl Radicals with 2-Methoxyphenol (Guaiacol) and Related Compounds, *J. Phys. Chem. A*, 2010, **114**, 11645–11650.

24 K. S. Docherty, a. C. Aiken, J. a. Huffman, I. M. Ulbrich, P. F. DeCarlo, D. Sueper, D. R. Worsnop, D. C. Snyder, R. E. Peltier, R. J. Weber, B. D. Grover, D. J. Eatough, B. J. Williams, a. H. Goldstein, P. J. Ziemann and J. L. Jimenez, The 2005 Study of Organic Aerosols at Riverside (SOAR-1): instrumental intercomparisons and fine particle composition, *Atmos. Chem. Phys.*, 2011, **11**, 12387–12420.

25 Y.-L. Sun, Q. Zhang, J. J. Schwab, K. L. Demerjian, W.-N. Chen, M.-S. Bae, H.-M. Hung, O. Hogrefe, B. Frank, O. V. Rattigan and Y.-C. Lin, Characterization of the sources and processes of organic and inorganic aerosols in New York city with a high-resolution time-of-flight aerosol mass apectrometer, *Atmos. Chem. Phys.*, 2011, **11**, 1581–1602.
